# Supplementary material for: Extreme weather events and dengue in Southeast Asia: A regionally-representative analysis of 291 locations from 1998 to 2021
Source: PLoS Negl Trop Dis. 2025 Sep 4;19(9):e0012649. doi: 10.1371/journal.pntd.0012649 (PMC12419652; doi:10.1371/journal.pntd.0012649)
Supplement: S1 Appendix — (DOCX) [file pntd.0012649.s001.docx]

**Appendix for manuscript**

**Extreme weather events and dengue in Southeast Asia: a regionally-representative analysis of 291 locations from 1998 to 2021**

# **S1 Appendix. Climate data collection**

Climate data was obtained from the ERA5-Land dataset generated by the European Centre for Medium-Range Weather Forecasts (ECMWF) through Google Earth Engine. The climate data collection begins by importing shapefiles of the Global Administrative Unit Layers 2015, First-Level Administrative Units from Food and Agriculture Organization (FAO) of the United Naitons (https://data.apps.fao.org/map/catalog/srv/eng/catalog.search?id=12691#/home), which provide detailed administrative boundaries corresponding to each study location. These shapefiles are uploaded to the Google Earth Engine platform using the 'Assets' tab, where they are stored as assets for use in the scripts. ERA5-Land hourly data and monthly data layers are accessed directly from the 'Datasets' available in Google Earth Engine. The specific meteorological variables required for the study, including mean temperature, dewpoint temperature, and precipitation are selected from the ERA5-Land collection. Each climate variable from ERA5-Land dataset is then precisely clipped to the designated administrative boundaries provided by the shapefiles, formatted into time series for data analysis.
