# Supplementary material for: Extreme weather events and dengue in Southeast Asia: A regionally-representative analysis of 291 locations from 1998 to 2021
Source: PLoS Negl Trop Dis. 2025 Sep 4;19(9):e0012649. doi: 10.1371/journal.pntd.0012649 (PMC12419652; doi:10.1371/journal.pntd.0012649)
Supplement: S1 Table — (DOCX) [file pntd.0012649.s002.docx]

# **S1 Table. Summary information of dengue data for each country.**

| **Country** | **Period** | **Number of locations** | **Case definition** | **Organization where data collected** |
| --- | --- | --- | --- | --- |
| Cambodia | 1998–2010 | 23 | Suspected dengue: very high fever at 39–40 degrees celcius for 2–7 days (usually 3–4 days), with 2 or more of the following signs: flushed face, headache, retro-orbital pain, myalgia/arthralgia, cutaneous rash, haemorrhagic signs (petechiae, positive tourniquet test), and leucopenia.  Probable dengue: signs of suspected dengue plus laboratory test results or that the case occurred in an area where the dengue case has been confirmed. | Communicable Disease Control (CDC) syndromic surveillance system (CamEWARN), Ministry of Health |
| Indonesia | 2010–2020 | 33 | Suspected dengue: Sudden high fever (usually ≥ 39°) plus two or more accompanying symptoms/signs:   - Headache - Pain behind the eyeballs - Muscle & bone pain - Skin rash - Bleeding manifestations - Leukopenia (Leukocytes ≤ 5000 /mm3) - Thrombocytopenia (platelets < 150,000 /mm3) - Increase in hematocrit 5 – 10% | Directorate of Communicable Diseases, Arbovirosis Teamwork, the Ministry of Health |
| Lao PDR | 2005–2021 | 2 | WHO dengue case classification (2009):  Any person who lived in, or travelled to, a dengue endemic area with onset of fever and two or more of the following:   - nausea/vomiting, - rash, - aches and pains, - tourniquet test positive, - leukopenia, **or** any warning sign. | National Centre for Laboratory and Epidemiology, Ministry of Health |
| Malaysia | 2010–2017 | 14 | **CASE DEFINITIONS FOR INFECTIOUS DISEASES IN MALAYSIA 3rd Ed (2017)**  **Clinical Case Definition**   1. *Dengue Fever:* Acute onset of high-grade fever of usually 2*–*5 days or more associated with two or more of the following: headache, retro-orbital pain, myalgia, arthralgia, rash and mild hemorrhagic manifestation (epistaxis, gums bleeding and petechiae). 2. *Dengue Hemorrhagic Fever:* A probable or confirmed case of Dengue Fever with hemorrhagic tendencies evidenced by one or more of the following:  - Positive tourniquet test (may be absent in pre-shock or shock state), - Petechiae, ecchymoses or purpura - Bleeding: mucosa, gastrointestinal tract (hematemesis, melena), injection sites and - Thrombocytopenia (100,000 cells per mm³ or less) - Evidence of plasma leakage due to increased vascular permeability: - Rise in hematocrit: ≥ 20% above baseline. - Signs of plasma leakage (pleural effusion and ascites, and /or hypoproteinemia).  1. *Dengue Shock Syndrome:* All the above criteria, plus evidence of circulatory failure manifested by rapid and weak pulse, and narrow pulse pressure (≤ 20 mm Hg) or hypotension for age, cold, clammy skin and altered mental status.   **Clinical Case Definition (Based on Warning Signs)**   1. *Dengue without Warning Signs:* Fever and two of the following:  - Nausea, vomiting - Rash | Disease Control Division, Ministry of Health Malaysia. |
| **Country** | **Period** | **Number of locations** | **Case definition** | **Organization where data collected** |
|  |  |  | - Aches and pains - Leukopenia - Positive tourniquet test  1. *Dengue with Warning Signs:* Dengue as defined above with any of the following:  - Abdominal pain or tenderness - Persistent vomiting - Clinical fluid accumulation (ascites, pleural effusion) - Mucosal bleeding - Lethargy, restlessness - Liver enlargement >2 cm - Laboratory: increase in HCT concurrent with rapid decrease in platelet count  1. *Severe Dengue:* Dengue with at least one of the following criteria:  - Severe Plasma Leakage leading to:   - Shock (DSS)   - Fluid accumulation with respiratory distress - Severe bleeding as evaluated by clinician - Severe organ involvement   - Liver: AST or ALT ≥ 1000   - NS: impaired consciousness   - Failure of heart and other organs  1. *Laboratory Criteria* (any of the following):  - Detection of Dengue Non-Structural Protein 1 (NS1) from serum. - Dengue IgM seroconversion in paired sera. - Dengue IgG seroconversion in paired sera or fourfold or greater rise dengue IgG in paired sera. - Detection of dengue virus genome in serum or CSF or biopsy samples by polymerase chain reaction (PCR). - Isolation of the dengue virus from serum, plasma, leukocytes, or biopsy samples. - Demonstration of dengue virus antigen in tissue biopsy by immunohistochemistry or immunofluorescence. - Detection of dengue IgM and /or IgG from/ in a single serum sample (highly suggestive). |  |
| The Philippines | 2010–2020 | 79 | WHO dengue case classification (2009):  Any person who lived in, or travelled to, a dengue endemic area with onset of fever and two or more of the following:   - nausea/vomiting, - rash, - aches and pains, - tourniquet test positive, - leukopenia, **or** any warning sign. | the Philippine Department of Health’s Epidemiological Bureau |
| Singapore | 2012–2019 | 1 | Fever, headache, backache, myalgia, rash, abdominal discomfort and thrombocytopenia | National Environment Agency and Ministry of Health |
| Thailand | 2003–2021 | 76 | Probable case: Fever, headache, backache, myalgia, rash, abdominal discomfort and thrombocytopenia. | the Department of Diseases Control, Ministry of Public Health |
| Vietnam | 2011–2021 | 63 | Acute onset of fever continuously lasting from 2–7 days AND at least 2 of the following: haemorrhagic manifestation /presentation; headache, loss of appetite, nausea, vomiting; rash; muscle pain, joint pain, orbital pain; lethargy; abdominal pain. | General Department of Preventive Medicine, Ministry of Health |
