# Supplementary material for: Extreme weather events and dengue in Southeast Asia: A regionally-representative analysis of 291 locations from 1998 to 2021
Source: PLoS Negl Trop Dis. 2025 Sep 4;19(9):e0012649. doi: 10.1371/journal.pntd.0012649 (PMC12419652; doi:10.1371/journal.pntd.0012649)
Supplement: S2 Table — (DOCX) [file pntd.0012649.s003.docx]

# **S2 Table. Example calculation of monthly number of heatwave days (HWt).**

Days where daily mean temperature (Tmean) exceeds the 95th percentile threshold, (30.5°C) are bolded and heatwave days (at least two consecutive days) are highlighted. Heatwaves that begin at the end of the preceding month and continue to the following month are accounted for.

| **Month** | **Week** | **Daily Mean Temperature/°C (95th percentile = 30.5°C)** | | | | | | | **Total No. of Heatwave Days**  $HW_{t}$ |
| --- | --- | --- | --- | --- | --- | --- | --- | --- | --- |
|  |  | **Sun** | **Mon** | **Tues** | **Wed** | **Thurs** | **Fri** | **Sat** |  |
| March | 4 | 30.0 | 29.7 | 29.0 | **31.0** | 28.9 | 30.0 | **32.4** |  |
| April | 1 | **30.7** | 29.0 | 30.1 | **30.7** | 29.8 | 30.3 | **33.5** | 2 |
| April | 2 | **33.5** | 29.8 | **33.8** | **33.9** | **33.5** | **33.4** | **33.9** | 6 |
| April | 3 | **33.8** | **34.0** | **33.5** | 30.3 | 30.4 | 28.7 | 29.0 | 3 |
| April | 4 | 28.9 | 29.0 | **30.7** | **30.9** | **32.7** | **32.3** | **32.4** | 5 |
|  | | | | | | | Total number of heatwave day per month | | 16 days |
