# Supplementary material for: Extreme weather events and dengue in Southeast Asia: A regionally-representative analysis of 291 locations from 1998 to 2021
Source: PLoS Negl Trop Dis. 2025 Sep 4;19(9):e0012649. doi: 10.1371/journal.pntd.0012649 (PMC12419652; doi:10.1371/journal.pntd.0012649)
Supplement: S3 Table — (DOCX) [file pntd.0012649.s004.docx]

# **S3 Table. Location-specific summary table of mean and standard deviation of each variable of each country.**

| **N** | **Province** | **Total dengue** | **Mean monthly temperature (SD)** | **Mean monthly total precipitation (SD)** | **Mean monthly relative humidity (SD)** | **Mean annual hw.2.95 (SD)** | **Mean annual hw.2.97 (SD)** | **Mean annual hw.2.99 (SD)** | **Mean annual hw.4.95 (SD)** | **Mean annual hw.4.97 (SD)** | **Mean annual hw.4.99 (SD)** | **Monthly mean scpdsi (SD)** |
| --- | --- | --- | --- | --- | --- | --- | --- | --- | --- | --- | --- | --- |
| 1 | Banteay Meanchey | 11402 | 27.4 (1.6) | 111.8 (87.9) | 70.5 (11.4) | 17.2 (19.3) | 9.5 (14.2) | 3.6 (7.7) | 11.7 (18) | 7 (12.7) | 2.2 (5.4) | 0.2 (2) |
| 2 | Battambang | 3491 | 26.8 (1.4) | 119 (84.6) | 73.7 (9.4) | 16.9 (19.8) | 9.3 (15.8) | 3.8 (8.8) | 10.8 (16.3) | 6.1 (13.1) | 2.5 (6.8) | 0.5 (1.8) |
| 3 | Kampong Cham | 21687 | 27.5 (1.5) | 140.5 (106.2) | 72.4 (12.8) | 17.7 (22.4) | 10.5 (15.1) | 3.3 (7.5) | 15.2 (21.1) | 8.1 (13.1) | 2.8 (7.3) | 0.3 (2.1) |
| 4 | Kampong Chhnang | 4524 | 27.4 (1.5) | 132.3 (97.2) | 72.6 (11.4) | 17.1 (23.3) | 10.3 (18.7) | 3.8 (10) | 13.4 (21.6) | 8 (17.1) | 2.9 (9.4) | 0.7 (2.2) |
| 5 | Kampong Thom | 7697 | 27.4 (1.5) | 141 (107.2) | 72.7 (13.1) | 17.1 (23) | 10.5 (17.5) | 3.2 (8.7) | 14 (22.1) | 7.4 (15.8) | 2.8 (8) | 1.2 (2.4) |
| 6 | Kampot | 3183 | 26 (1) | 159.1 (105.2) | 79.4 (8.7) | 17.5 (23.3) | 9.4 (15) | 3.4 (7.9) | 13.2 (21.3) | 7.1 (13.4) | 1.9 (6.9) | -0.2 (2.2) |
| 7 | Kandal | 25723 | 27.6 (1.3) | 126.4 (94.6) | 72.9 (10.1) | 17.6 (21.7) | 10.4 (14.1) | 3.2 (6.1) | 14.2 (18.7) | 7.2 (11.5) | 2.5 (6.2) | -0.5 (2) |
| 8 | Kep | 549 | 26.8 (0.9) | 142.6 (95.9) | 79.4 (7.1) | 17.1 (22.1) | 9.3 (15) | 3.2 (7.4) | 12.5 (20.1) | 6.8 (13.6) | 2.1 (6.4) | -0.4 (2.3) |
| 9 | Koh Kong | 603 | 24.7 (0.8) | 240.8 (164.3) | 83.9 (8.8) | 16.2 (28.5) | 9.9 (21.2) | 3.5 (8.9) | 12.8 (26.9) | 7.5 (19.3) | 1.8 (6.7) | 0.5 (2.1) |
| 10 | Kratie | 1303 | 27.2 (1.6) | 154.5 (123.4) | 72.6 (15) | 17.5 (21.2) | 10.9 (15.9) | 3.2 (7.9) | 13.8 (19.9) | 8.2 (15.5) | 2.6 (6.7) | 0.9 (2.1) |
| 11 | Mondul Kiri | 35 | 26 (1.6) | 153.3 (126.8) | 72.4 (14.4) | 18.1 (21) | 10.3 (14) | 3.2 (6.5) | 14.1 (19) | 7.5 (12) | 2.1 (5.1) | 0.6 (1.9) |
| 12 | Otdar Meanchey | 1696 | 27 (1.7) | 112.1 (93.4) | 70.7 (12.2) | 16.5 (16.8) | 10.2 (13.9) | 3.7 (8.6) | 12 (14.3) | 7.4 (12.9) | 2.5 (6.9) | 1 (2.5) |
| 13 | Pailin | 494 | 25.2 (1.1) | 150 (105.6) | 80.3 (9.8) | 15.5 (21.1) | 9.2 (17.3) | 3.4 (8.8) | 10 (19.3) | 6.2 (14.4) | 2.7 (8.6) | -0.1 (1.8) |
| 14 | Phnom Penh | 24232 | 27.7 (1.4) | 119 (90.5) | 72 (10.2) | 16.5 (23.2) | 9.2 (15.4) | 3.3 (8.8) | 12.2 (19.6) | 5.7 (12.2) | 2.2 (8) | -0.1 (2.2) |
| 15 | Preah Sihanouk | 1260 | 26.1 (0.8) | 266.3 (190.2) | 82.9 (7.5) | 16.1 (23.9) | 10.3 (19.7) | 3.4 (8.1) | 12.6 (21.9) | 7.8 (17.5) | 1.9 (6.9) | 0.5 (2.6) |
| 16 | Preah Vihear | 990 | 26.8 (1.6) | 142.6 (115.6) | 73.3 (13.8) | 17.5 (19.1) | 10.2 (16.1) | 3.4 (10.8) | 13.4 (18.1) | 5.9 (15.9) | 2.6 (9.4) | 1.4 (2.4) |
| 17 | Prey Veng | 6958 | 27.6 (1.3) | 135.6 (100.8) | 73 (10.6) | 17.5 (20.3) | 10.5 (13.4) | 3.2 (5.1) | 15.5 (18.3) | 7.2 (10.4) | 2.4 (4.4) | -0.9 (1.9) |
| 18 | Pursat | 1817 | 25.5 (1.3) | 159.1 (103.9) | 77.9 (9) | 17.2 (22.6) | 9.2 (17) | 3.8 (9.6) | 13.5 (20.7) | 7.2 (15) | 2.8 (8.9) | 0.7 (2) |
| 19 | Ratanak Kiri | 542 | 26.1 (1.6) | 180.7 (162.4) | 72.9 (15.7) | 18.2 (18.7) | 9.9 (13.2) | 3.1 (6.2) | 12.2 (16.4) | 6.6 (12) | 2.2 (5.6) | 0.9 (2.2) |
| 20 | Siem Reap | 20196 | 27.4 (1.5) | 129.6 (98.5) | 72.1 (11.1) | 17.3 (19.9) | 10.2 (15.3) | 3.6 (9.7) | 12.7 (18.8) | 6.2 (13.8) | 3 (8.5) | 1 (2.4) |
| 21 | Stung Treng | 507 | 26.8 (1.6) | 175.4 (153.4) | 73.3 (15.7) | 17.3 (19.8) | 10.3 (15.5) | 3.2 (8.2) | 13.3 (18.7) | 6.8 (14.6) | 2.5 (6.5) | 1.4 (2.3) |
| 22 | Svay Rieng | 2226 | 27.4 (1.3) | 149.8 (112.2) | 74.2 (10.6) | 17.9 (20.5) | 10.5 (13) | 3.2 (5) | 15.8 (17.8) | 7.8 (11.1) | 2.4 (4.4) | -2 (1.6) |
| 23 | Takeo | 10943 | 27.2 (1.2) | 137.1 (98.3) | 75.3 (9.4) | 17.2 (23.7) | 9.5 (15.5) | 3.4 (7.9) | 13.1 (21.3) | 6.8 (13.4) | 2.2 (6.9) | -0.8 (1.9) |

1. Cambodia

Note: SD: standard deviation; hw.2.95: heatwave with 95^th^ percentile for 2 consecutive days; hw.2.97: heatwave with 97^th^ percentile for 2 consecutive days; hw.2.99: heatwave with 99^th^ percentile for 2 consecutive days; hw.4.95: heatwave with 95^th^ percentile for 4 consecutive days; hw.4.97: heatwave with 97^th^ percentile for 4 consecutive days; hw.4.99: heatwave with 99^th^ percentile for 4 consecutive days; scPDSI: self-calibrated Palmer Drought Severity Index.

2. Indonesia

| **N** | **Province** | **Total dengue** | **Mean monthly temperature (SD)** | **Mean monthly total precipitation (SD)** | **Mean monthly relative humidity (SD)** | **Mean annual hw.2.95 (SD)** | **Mean annual hw.2.97 (SD)** | **Mean annual hw.2.99 (SD)** | **Mean annual hw.4.95 (SD)** | **Mean annual hw.4.97 (SD)** | **Mean annual hw.4.99 (SD)** | **Monthly mean scpdsi (SD)** |
| --- | --- | --- | --- | --- | --- | --- | --- | --- | --- | --- | --- | --- |
| 1 | Aceh | 22809 | 22.6 (0.5) | 287.4 (111.2) | 87.2 (3.1) | 14.8 (13.6) | 8.2 (7.7) | 2.7 (3) | 8.5 (8) | 4 (4.3) | 0.9 (2.1) | 0.5 (1.6) |
| 2 | Bali | 87503 | 24.2 (0.6) | 228.3 (176.3) | 84.5 (4.8) | 13.5 (18.5) | 8.6 (12.2) | 2.1 (3.4) | 7.9 (13) | 4.5 (7.3) | 0.5 (1.5) | -1 (1.9) |
| 3 | Bangka Belitung | 7334 | 26.7 (0.4) | 229 (104.3) | 84.5 (4.1) | 13 (12.1) | 6.7 (7.2) | 1.7 (3.2) | 8.3 (9.8) | 4.3 (6.8) | 0.8 (1.8) | -0.6 (2) |
| 4 | Banten | 34979 | 25.6 (0.4) | 227.8 (133.5) | 84.4 (4.4) | 13.5 (16.2) | 7.5 (10.3) | 3.1 (5.2) | 8.5 (12.8) | 5.2 (8.6) | 1.7 (3.3) | 0 (2.2) |
| 5 | Bengkulu | 12818 | 23.5 (0.4) | 304.7 (116.8) | 87.6 (2.3) | 14.6 (15.1) | 7.7 (11.3) | 2.7 (7.1) | 7.9 (13.8) | 4.1 (9.2) | 1.5 (5.1) | -0.6 (1.7) |
| 6 | Di Yogyakarta | 30999 | 24.9 (0.6) | 202.8 (153.3) | 84.2 (4.2) | 17.2 (18.4) | 9.9 (12.8) | 3 (4.8) | 13.5 (17.3) | 7.2 (11.1) | 1.5 (3.4) | 0.2 (1.4) |
| 7 | Dki Jakarta | 96340 | 26.5 (0.6) | 178.7 (115.5) | 80.9 (6.1) | 13.9 (18.6) | 8.5 (12.3) | 2.9 (5.2) | 9.3 (15.4) | 5.4 (9.1) | 1.8 (3.5) | 0.4 (1.9) |
| 8 | Gorontalo | 5629 | 24.4 (0.4) | 289.5 (106.2) | 87.1 (4.3) | 14 (15.5) | 7.5 (9.2) | 2.3 (3.6) | 9.5 (13.4) | 5.2 (9.4) | 1.4 (2.4) | -0.7 (1.7) |
| 9 | Jambi | 13356 | 25 (0.5) | 248.9 (91.9) | 85.9 (4.1) | 15.8 (20.3) | 9.7 (16.5) | 3.1 (6.9) | 9.9 (18.6) | 7.4 (13.8) | 2.2 (4.9) | -0.2 (1.6) |
| 10 | Java Central | 113766 | 24.9 (0.5) | 244.1 (163.1) | 82.9 (7.2) | 17 (19.4) | 9.9 (12.9) | 2.8 (5.9) | 13.5 (19.3) | 6.2 (11.4) | 2 (5.2) | 0.1 (1.4) |
| 11 | Java East | 151635 | 24.9 (0.6) | 202.1 (152.7) | 81.6 (6.7) | 16.3 (16.1) | 8.7 (10.6) | 2.2 (3.4) | 13.5 (16.6) | 5 (8.1) | 0.8 (1.8) | -0.1 (2) |
| 12 | Java West | 223307 | 23.9 (0.4) | 254.4 (151.5) | 83.9 (6.3) | 15.5 (16.9) | 8.9 (11.9) | 3.3 (6) | 10.5 (14.8) | 5.9 (9.8) | 2.7 (4.9) | 0 (1.6) |
| 13 | Kalimantan Central | 13986 | 25.7 (0.4) | 260.3 (88.9) | 86.1 (4.4) | 13.8 (15.5) | 7.5 (11.9) | 2.4 (4.4) | 8.5 (14.6) | 4.8 (8.4) | 1.2 (2.6) | -0.3 (1.3) |
| 14 | Kalimantan East | 50271 | 24.3 (0.4) | 270 (64.9) | 88.1 (2.5) | 12.9 (13) | 6.9 (8.1) | 2.1 (3.5) | 5.5 (9.9) | 2.8 (5.7) | 1.3 (2.8) | 1 (1.8) |
| 15 | Kalimantan South | 19454 | 25.8 (0.5) | 226 (100.3) | 85.8 (5.2) | 14.3 (17.5) | 8.1 (12.9) | 2.6 (6.1) | 9.2 (15) | 6.3 (12.1) | 1.5 (3.8) | 0.1 (1.5) |
| 16 | Kalimantan West | 19795 | 25.3 (0.4) | 273.9 (86) | 88.2 (3.1) | 11.7 (10.2) | 6.6 (7.2) | 1.9 (3.5) | 5.2 (7.7) | 2.8 (5.2) | 0.8 (2.7) | -0.3 (1.7) |
| 17 | Kepulauan Riau | 15846 | 27 (0.6) | 213.2 (90.7) | 82.6 (2.1) | 14.5 (7.2) | 8 (4.5) | 2.4 (2.8) | 8.5 (6.1) | 2.5 (3.1) | 0.5 (1.5) | 0.8 (2.1) |
| 18 | Lampung | 37209 | 25.4 (0.5) | 223.7 (109.3) | 85 (5.2) | 15.5 (21.8) | 9 (16.1) | 3.5 (6.8) | 12.5 (19.2) | 7 (13.8) | 2.5 (5.5) | 0.2 (1.8) |
| 19 | Maluku | 1331 | 25.1 (0.6) | 238.1 (96.3) | 86 (2.7) | 14.8 (12.2) | 8.6 (9.7) | 2.5 (3.8) | 7.5 (9.2) | 3.7 (6.1) | 1.7 (3.9) | 0.1 (0.5) |
| 20 | Maluku North | 3149 | 24.8 (0.4) | 283 (104.6) | 86.7 (2.4) | 15.5 (13.8) | 8.3 (9.3) | 2.7 (5.7) | 7.4 (8.3) | 3.7 (6.9) | 1.4 (3.4) | -0.1 (1.1) |
| 21 | Nusa Tenggara East | 16605 | 24.8 (0.9) | 149.4 (127.6) | 79.5 (7.2) | 17 (13) | 9.9 (10.5) | 2.8 (4.8) | 13.7 (13.1) | 5.9 (9.8) | 2.5 (4.9) | -0.3 (1.6) |
| 22 | Nusa Tenggara West | 19625 | 24.9 (0.7) | 166.6 (141.9) | 81.9 (5.5) | 15.2 (13.1) | 9 (10.3) | 2.9 (5.4) | 11.9 (12.7) | 6.8 (11.2) | 1.4 (4.5) | -0.7 (1.9) |
| 23 | Papua | 4138 | 23.4 (0.4) | 401.8 (70.2) | 88.1 (2.1) | 11.8 (9.2) | 6.9 (6.1) | 2.1 (2.2) | 4.3 (5.9) | 2.5 (3.8) | 0.8 (1.8) | 0 (1.2) |
| 24 | Papua West | 1514 | 24.2 (0.4) | 322.9 (79) | 89 (1.9) | 11.7 (12.6) | 6.4 (8.5) | 2.4 (4.7) | 4.3 (7.9) | 2.9 (5.6) | 0.8 (2.7) | 0.5 (1.4) |
| 25 | Riau | 26151 | 26 (0.5) | 222 (80.1) | 87.4 (2.9) | 13.8 (8.8) | 7.7 (6) | 2.4 (3.1) | 7.3 (6.3) | 3 (3.7) | 0.5 (1.8) | 0.3 (1.7) |
| 26 | Southeast Sulawesi | 12315 | 24.5 (0.7) | 246.8 (114.8) | 85.1 (5.5) | 15.5 (13.6) | 9.6 (11.1) | 3.3 (7.5) | 11.2 (13.4) | 5.9 (10.6) | 2.7 (6.5) | 0.5 (1.1) |
| 27 | Sulawesi Central | 18405 | 22.9 (0.4) | 294.3 (89.5) | 87.7 (3.1) | 12.5 (14.5) | 7.3 (11) | 1.9 (4) | 7.2 (11) | 3.6 (6.3) | 0.4 (1.2) | 0.5 (1.1) |
| 28 | Sulawesi North | 14624 | 24.1 (0.4) | 275.6 (102.4) | 87.3 (3.5) | 12.8 (13.4) | 7.5 (10.3) | 2.4 (4.8) | 8.3 (10.9) | 4.9 (8.6) | 0.8 (1.8) | -0.9 (1.8) |
| 29 | Sulawesi South | 36588 | 23.4 (0.6) | 280.9 (119.8) | 84.3 (5.4) | 13.7 (15.9) | 8.2 (13.3) | 3 (6.9) | 8.9 (15.2) | 5.4 (11.7) | 1.8 (4.1) | 1.1 (1.2) |
| 30 | Sulawesi West | 5169 | 22.2 (0.3) | 334.1 (114.3) | 88.4 (2.5) | 10.8 (9.4) | 6.2 (6) | 1.6 (2.1) | 2.7 (4) | 1.8 (3.7) | 0.6 (2.1) | 1.4 (1.7) |
| 31 | Sumatera North | 63922 | 23.7 (0.5) | 270.1 (94.3) | 87.5 (2.7) | 12.9 (7.9) | 7.5 (5.3) | 1.7 (1.8) | 4.8 (4.2) | 3.5 (3.6) | 0.4 (1.2) | -0.1 (2.3) |
| 32 | Sumatera South | 25613 | 25.7 (0.7) | 232 (104.4) | 84.3 (6.5) | 17.3 (23.5) | 10.1 (15.9) | 3.4 (5.7) | 15.6 (22.3) | 8 (12.2) | 2.7 (4.3) | -0.4 (2) |
| 33 | Sumatera West | 27533 | 23.5 (0.4) | 310.7 (109.8) | 87.7 (2.2) | 12.9 (10.7) | 6.8 (7.9) | 2 (2.5) | 5.7 (6) | 3.2 (3.3) | 0.4 (1.2) | 0.5 (1.7) |

Note: SD: standard deviation; hw.2.95: heatwave with 95^th^ percentile for 2 consecutive days; hw.2.97: heatwave with 97^th^ percentile for 2 consecutive days; hw.2.99: heatwave with 99^th^ percentile for 2 consecutive days; hw.4.95: heatwave with 95^th^ percentile for 4 consecutive days; hw.4.97: heatwave with 97^th^ percentile for 4 consecutive days; hw.4.99: heatwave with 99^th^ percentile for 4 consecutive days; scPDSI: self-calibrated Palmer Drought Severity Index.

3. LAO PDR

| **N** | **Province** | **Total dengue** | **Mean monthly temperature (SD)** | **Mean monthly total precipitation (SD)** | **Mean monthly relative humidity (SD)** | **Mean annual hw.2.95 (SD)** | **Mean annual hw.2.97 (SD)** | **Mean annual hw.2.99 (SD)** | **Mean annual hw.4.95 (SD)** | **Mean annual hw.4.97 (SD)** | **Mean annual hw.4.99 (SD)** | **Monthly mean scpdsi (SD)** |
| --- | --- | --- | --- | --- | --- | --- | --- | --- | --- | --- | --- | --- |
| 1 | Savannakhet | 21250 | 24.7 (2.4) | 148.1 (149.1) | 76.6 (9.1) | 16.9 (15.4) | 10.2 (10.6) | 3.3 (4.5) | 10.9 (13) | 7.2 (9.9) | 1.2 (3) | -0.3 (1.9) |
| 2 | Vientiane capital | 42141 | 25.5 (2.3) | 167.3 (168.4) | 73.6 (11.5) | 17.4 (11.7) | 10.2 (10) | 3.2 (4.6) | 11.5 (12) | 5.8 (8.4) | 1.6 (3) | 0.5 (2.3) |

Note: SD: standard deviation; hw.2.95: heatwave with 95^th^ percentile for 2 consecutive days; hw.2.97: heatwave with 97^th^ percentile for 2 consecutive days; hw.2.99: heatwave with 99^th^ percentile for 2 consecutive days; hw.4.95: heatwave with 95^th^ percentile for 4 consecutive days; hw.4.97: heatwave with 97^th^ percentile for 4 consecutive days; hw.4.99: heatwave with 99^th^ percentile for 4 consecutive days; scPDSI: self-calibrated Palmer Drought Severity Index.

| **N** | **Province** | **Total dengue** | **Mean monthly temperature (SD)** | **Mean monthly total precipitation (SD)** | **Mean monthly relative humidity (SD)** | **Mean annual hw.2.95 (SD)** | **Mean annual hw.2.97 (SD)** | **Mean annual hw.2.99 (SD)** | **Mean annual hw.4.95 (SD)** | **Mean annual hw.4.97 (SD)** | **Mean annual hw.4.99 (SD)** | **Monthly mean scpdsi (SD)** |
| --- | --- | --- | --- | --- | --- | --- | --- | --- | --- | --- | --- | --- |
| 1 | Johor | 53106 | 26 (0.6) | 222.6 (94.3) | 87 (2.9) | 13.6 (14.8) | 8.1 (9.4) | 2.6 (5.1) | 8.3 (9.6) | 4.1 (7.1) | 1.3 (3.5) | 1.1 (2) |
| 2 | Kedah | 7364 | 25.7 (0.9) | 203.9 (97.9) | 83.7 (6.9) | 18.1 (26.3) | 11.1 (20.6) | 3.4 (9.5) | 14.5 (24.9) | 9 (19.1) | 3.1 (8.8) | -0.5 (1.8) |
| 3 | Kelantan | 32994 | 24.5 (1.1) | 212.9 (93) | 85.5 (5.5) | 16.8 (26.8) | 10.5 (21.3) | 3.6 (10.3) | 14 (24.9) | 7.9 (18.2) | 2.4 (6.7) | -1.2 (1.7) |
| 4 | Kuala Lumpur | 43157 | 26 (0.7) | 239.7 (107) | 85.5 (3.9) | 13.6 (17.9) | 8.6 (13.7) | 2.8 (5) | 8.3 (12.6) | 5.8 (9.2) | 1.4 (2.6) | -0.1 (1.7) |
| 5 | Melaka | 12830 | 26.5 (0.7) | 188.3 (91) | 83.4 (3.7) | 14.5 (22.4) | 8.6 (17.1) | 3.1 (8.1) | 9.5 (18.4) | 7.4 (15.9) | 2.3 (6.4) | 0.7 (2.1) |
| 6 | Negeri Sembilan | 16130 | 25.6 (0.8) | 197.7 (87.5) | 85.4 (4.3) | 16 (22.4) | 8.8 (18.4) | 3.6 (10.3) | 9.5 (19.1) | 6.5 (16.8) | 3 (8.5) | 0.4 (1.8) |
| 7 | Pahang | 13859 | 25.3 (0.9) | 212.9 (88.3) | 85.1 (4.6) | 16.4 (25.1) | 9.6 (18.4) | 3.4 (9.5) | 10.8 (20.7) | 5.9 (13.5) | 2.3 (6.4) | -0.4 (1.6) |
| 8 | Perak | 34113 | 24.6 (0.8) | 223.2 (94.7) | 84.9 (4.2) | 15.5 (25.7) | 9.8 (20.4) | 4 (11.3) | 11 (21.7) | 7 (16.8) | 3.3 (9.2) | -0.9 (2.1) |
| 9 | Perlis | 1656 | 26.7 (0.9) | 174.7 (90.6) | 81.4 (7.3) | 19 (25.3) | 10.5 (20) | 3.6 (10.3) | 15.9 (23.7) | 8.9 (18.5) | 1.9 (5.3) | 0 (1.6) |
| 10 | Pulau Pinang | 19485 | 26.5 (0.7) | 216.5 (103) | 83.1 (4.2) | 16.6 (25.2) | 10.6 (20.1) | 3.3 (8.4) | 11.8 (21.9) | 8.3 (18.1) | 3 (8.5) | -0.9 (2.2) |
| 11 | Sabah | 15049 | 24.4 (0.6) | 232.6 (66.5) | 87.8 (2.6) | 15 (24.7) | 8.3 (16) | 3 (8.5) | 9.9 (20.5) | 5.3 (11.8) | 1.5 (4.2) | 0.4 (2.1) |
| 12 | Sarawak | 15286 | 24.4 (0.5) | 298.1 (85.1) | 88.6 (1.6) | 9.6 (6.2) | 5.3 (4.1) | 1.3 (1.8) | 2.5 (2.8) | 0.6 (1.8) | 0 (0) | -0.8 (1.6) |
| 13 | Selangor | 271520 | 25.9 (0.7) | 216.9 (96.4) | 84.6 (3.8) | 13.4 (15.9) | 8.8 (12.1) | 2.1 (3.4) | 9.1 (12.5) | 5.4 (7) | 1.1 (2.1) | -0.2 (1.8) |
| 14 | Terengganu | 8930 | 25.2 (0.9) | 264.9 (115.5) | 87.4 (3.4) | 16 (20) | 10.9 (16.3) | 3.9 (9.1) | 13 (18.8) | 9.6 (15) | 3.4 (7.7) | -1 (1.5) |

4. Malaysia

Note: SD: standard deviation; hw.2.95: heatwave with 95^th^ percentile for 2 consecutive days; hw.2.97: heatwave with 97^th^ percentile for 2 consecutive days; hw.2.99: heatwave with 99^th^ percentile for 2 consecutive days; hw.4.95: heatwave with 95^th^ percentile for 4 consecutive days; hw.4.97: heatwave with 97^th^ percentile for 4 consecutive days; hw.4.99: heatwave with 99^th^ percentile for 4 consecutive days; scPDSI: self-calibrated Palmer Drought Severity Index.

5. Philippines

| **N** | **Province** | **Total dengue** | **Mean monthly temperature (SD)** | **Mean monthly total precipitation (SD)** | **Mean monthly relative humidity (SD)** | **Mean annual hw.2.95 (SD)** | **Mean annual hw.2.97 (SD)** | **Mean annual hw.2.99 (SD)** | **Mean annual hw.4.95 (SD)** | **Mean annual hw.4.97 (SD)** | **Mean annual hw.4.99 (SD)** | **Monthly mean scpdsi (SD)** |
| --- | --- | --- | --- | --- | --- | --- | --- | --- | --- | --- | --- | --- |
| 1 | Abra | 7163 | 22.7 (1.2) | 235.6 (200.5) | 82.1 (5.2) | 15.4 (7.6) | 9 (6.7) | 2.5 (3.7) | 11.1 (8.2) | 6.3 (6.4) | 1.7 (3.2) | 0 (1.4) |
| 2 | Agusan Del Norte | 16735 | 24.8 (0.7) | 232.8 (138.8) | 83.7 (3) | 15 (16.1) | 8.6 (9.1) | 2.4 (3.7) | 11.5 (12.5) | 4.2 (5.7) | 0.9 (2) | 1.1 (1.8) |
| 3 | Agusan Del Sur | 15012 | 25.2 (0.7) | 248.2 (106.1) | 85.1 (2.7) | 15 (18.4) | 7.9 (9.9) | 2 (3.1) | 9.4 (13.2) | 4 (6) | 0.4 (1.2) | 1.2 (2) |
| 4 | Aklan | 17484 | 25.4 (0.8) | 242.5 (121.3) | 85 (2.8) | 16.9 (16.8) | 10.4 (13) | 3.5 (4.8) | 13.5 (15.8) | 9.1 (12.2) | 2.6 (3.8) | 2 (1.5) |
| 5 | Albay | 6566 | 25.9 (1) | 236.8 (137.1) | 84.1 (3.9) | 16.6 (14.3) | 9.1 (9.8) | 3.1 (4.5) | 11.7 (11.5) | 6.5 (7.9) | 2.3 (4) | 1.3 (1.8) |
| 6 | Antique | 11929 | 25 (0.9) | 269.2 (169.1) | 83.6 (7.1) | 17.4 (18) | 10.6 (13.3) | 3.2 (5.5) | 15.8 (17.7) | 8.7 (12.3) | 2.8 (5.5) | 2.2 (1.2) |
| 7 | Apayao | 6527 | 23 (2) | 280.3 (148.6) | 86.4 (3.1) | 15.7 (11.2) | 8.5 (8.3) | 3.1 (3.7) | 9.8 (9.5) | 5.2 (5.4) | 2.5 (3.6) | -0.1 (1.4) |
| 8 | Aurora | 6189 | 23.7 (1.4) | 239.5 (134.9) | 85.6 (1.8) | 17.5 (10.4) | 10.1 (8.3) | 3.4 (4.4) | 12 (9.3) | 6.9 (7.3) | 2.3 (3.3) | -0.6 (1.5) |
| 9 | Basilan | 6362 | 26.1 (0.5) | 185.4 (87) | 83.9 (3.7) | 13.2 (13.3) | 7.6 (9.6) | 2.6 (6.8) | 5.9 (9.2) | 4.5 (8.3) | 1.1 (3.6) | 0.5 (2.2) |
| 10 | Bataan | 21344 | 26.1 (1.1) | 210.4 (214.4) | 79.1 (6.7) | 18.5 (11.2) | 10.9 (8.4) | 2.5 (3.4) | 15 (11.3) | 8 (8.6) | 1.3 (2.9) | 0.1 (1.4) |
| 11 | Batangas | 63424 | 26.2 (1.1) | 199.5 (162.7) | 81.2 (5.7) | 17.8 (11.9) | 10.4 (9.5) | 3.5 (5.2) | 14.5 (11.9) | 8.4 (9.7) | 2.4 (3.5) | 0.6 (1.8) |
| 12 | Benguet | 27384 | 20.6 (1) | 295.3 (232.3) | 84.8 (4.4) | 15.9 (13.3) | 9.2 (9.2) | 2.9 (5) | 10 (11.1) | 5.2 (6.8) | 2 (3.5) | -0.3 (1.5) |
| 13 | Biliran | 2763 | 26.2 (0.7) | 209 (146.5) | 80.8 (2.5) | 17.4 (15.6) | 9.8 (10.5) | 2.9 (5.1) | 14.5 (14.5) | 6.3 (8.6) | 2.1 (4.7) | 1.5 (1.8) |
| 14 | Bohol | 30469 | 26.2 (0.8) | 226.1 (109.6) | 83.6 (3.5) | 17 (14.6) | 10 (9.4) | 3.4 (4.7) | 13.5 (12.2) | 6.9 (7.6) | 1.7 (4.3) | 2.1 (1.6) |
| 15 | Bukidnon | 37693 | 22.3 (0.6) | 254.9 (108.5) | 85.7 (3.7) | 16.2 (21.4) | 9.2 (16.3) | 2.9 (5.8) | 10.9 (17.9) | 5.5 (12.8) | 1.6 (4.1) | 1.5 (1.9) |
| 16 | Bulacan | 68472 | 25.7 (1.2) | 211.1 (160.7) | 81.4 (5.9) | 17.6 (12.3) | 10.5 (9.1) | 3.3 (4) | 15.2 (11.8) | 7.9 (8.8) | 2.3 (3.2) | 0.1 (1.6) |
| 17 | Cagayan | 33503 | 25 (2) | 252.8 (143.1) | 84.1 (3.1) | 15.1 (10.7) | 8.2 (8.3) | 3 (3.3) | 9.2 (9.1) | 5.1 (5.3) | 2.3 (3.4) | -0.5 (1.5) |
| 18 | Camarines Norte | 3059 | 26.1 (1.1) | 227.5 (123.5) | 83.8 (3.4) | 17.4 (12.5) | 9.5 (8.2) | 3 (3.5) | 11.4 (11.1) | 6.5 (6.8) | 1.3 (2.2) | 0.2 (1.8) |
| 19 | Camarines Sur | 18540 | 26 (1) | 226.6 (126.8) | 83.8 (3.4) | 16.9 (13.8) | 8.9 (9.5) | 3.5 (4.4) | 11.5 (12.2) | 6.3 (8) | 2.4 (3.7) | 1.5 (1.6) |
| 20 | Camiguin | 3507 | 26.1 (0.7) | 165.7 (122.1) | 81.5 (2.1) | 17.8 (15.8) | 9.9 (9.8) | 3.5 (4.5) | 14.2 (13.1) | 7.9 (8.4) | 1.6 (4.1) | 1.5 (1.8) |
| 21 | Capiz | 19721 | 25.5 (0.8) | 271.1 (121.6) | 85.7 (3.1) | 16.9 (16.8) | 10.5 (13.3) | 3.5 (5.3) | 13.5 (15.8) | 9.1 (12.1) | 2.6 (5) | 2.6 (1.1) |
| **N** | **Province** | **Total dengue** | **Mean monthly temperature (SD)** | **Mean monthly total precipitation (SD)** | **Mean monthly relative humidity (SD)** | **Mean annual hw.2.95 (SD)** | **Mean annual hw.2.97 (SD)** | **Mean annual hw.2.99 (SD)** | **Mean annual hw.4.95 (SD)** | **Mean annual hw.4.97 (SD)** | **Mean annual hw.4.99 (SD)** | **Monthly mean scpdsi (SD)** |
| 22 | Catanduanes | 2425 | 26.1 (1) | 218.6 (117.5) | 82.6 (3.1) | 16.4 (11.7) | 10 (8) | 2.5 (4) | 11.3 (11.4) | 6.8 (7.5) | 1.3 (3) | 1.3 (1.7) |
| 23 | Cavite | 78013 | 26.2 (1.1) | 220.3 (191.1) | 80.4 (6.4) | 18.3 (11.2) | 10.7 (8.1) | 2.7 (3.4) | 14.1 (10.8) | 8.4 (8.3) | 1.3 (2.9) | 0.4 (1.8) |
| 24 | Cebu | 117065 | 26 (0.7) | 200.9 (98.2) | 82.3 (2.5) | 17.8 (17.3) | 10 (11.1) | 3.4 (4.9) | 14.5 (15.7) | 7.9 (9.9) | 2.4 (4.1) | 2.4 (1.2) |
| 25 | Davao del Norte | 18325 | 25.4 (0.7) | 204.3 (85.9) | 82.7 (3.1) | 16.1 (22.1) | 9.8 (16.6) | 2.8 (6.3) | 12.7 (21.3) | 7.5 (14.7) | 1.8 (4) | 1.5 (2.1) |
| 26 | Davao Del Sur | 51716 | 24.4 (0.5) | 209.2 (88.1) | 83.5 (3.7) | 14.3 (12.6) | 8.2 (8) | 2.2 (3.2) | 8.5 (10.4) | 3.2 (6.2) | 1.2 (2.9) | 1.4 (2) |
| 27 | Davao Oriental | 10126 | 24.4 (0.5) | 268 (103.4) | 86.3 (1.8) | 14.8 (19.6) | 9.8 (17) | 3 (6.7) | 9.9 (17.5) | 7 (14.7) | 1.7 (3.8) | 1.4 (2) |
| 28 | Dinagat | 1823 | 26.5 (0.7) | 241.1 (164.3) | 82.2 (2.6) | 15.1 (11.1) | 9.4 (7.5) | 2.9 (3.4) | 9.7 (7.9) | 6 (5.1) | 0.9 (2) | 0.8 (1.7) |
| 29 | Eastern Samar | 12272 | 25.9 (0.8) | 239.5 (134) | 85.2 (2.8) | 16.1 (10.7) | 9.6 (8.7) | 2.9 (4.1) | 9.6 (9.9) | 5.5 (7.6) | 1.6 (2.9) | 1.2 (1.7) |
| 30 | Guimaras | 5306 | 26.7 (0.9) | 169.7 (122) | 81.2 (5.1) | 17.4 (18.1) | 10.7 (14.2) | 3.4 (6) | 15.6 (18) | 8.4 (12.9) | 2.5 (5.7) | 2 (1.5) |
| 31 | Ifugao | 8606 | 21.7 (1.7) | 265.7 (141.3) | 84.8 (2.8) | 15.1 (12.4) | 9 (9.1) | 2.2 (3.4) | 8.6 (11) | 4.5 (5.9) | 0.9 (2.1) | -0.3 (1.7) |
| 32 | Ilocos Norte | 16661 | 24.5 (1.4) | 218.4 (203.1) | 80.8 (5.3) | 15.7 (7.1) | 8.5 (4.9) | 2.5 (3.1) | 12.5 (5.5) | 5.4 (5.3) | 1.5 (2.7) | 0.2 (1.5) |
| 33 | Ilocos Sur | 22864 | 23.9 (0.9) | 271 (243.9) | 82 (4.1) | 15.3 (8.8) | 8.6 (7.1) | 3 (4.3) | 11.3 (9.2) | 6.2 (6.4) | 1.6 (3) | 0.1 (1.4) |
| 34 | Iloilo | 75018 | 25.7 (0.8) | 230.6 (116.9) | 84.6 (4.4) | 17.5 (18.2) | 10.5 (13) | 3.2 (6.1) | 16 (17.9) | 8.7 (11.9) | 2.5 (5.8) | 2.4 (1.2) |
| 35 | Isabela | 36717 | 24.6 (1.9) | 239.9 (132.2) | 84.4 (3.6) | 15.5 (12.1) | 8.4 (7.5) | 2.2 (3.6) | 8.8 (10.4) | 3.7 (4.9) | 1.1 (2.4) | -0.3 (1.5) |
| 36 | Kalinga | 15535 | 22.1 (1.8) | 263.6 (135.4) | 85.6 (3.1) | 15.5 (10) | 8.5 (7.8) | 2.5 (3.9) | 8.5 (8.2) | 4.7 (5.6) | 2 (3.9) | -0.2 (1.5) |
| 37 | La Union | 27365 | 25.4 (1) | 271.4 (235.1) | 83.8 (2.9) | 15.9 (13.3) | 9.2 (9.2) | 2.9 (5) | 10 (11.1) | 5.2 (6.8) | 2 (3.5) | -0.1 (1.4) |
| 38 | Laguna | 80387 | 25.3 (1.2) | 211.3 (145.1) | 83.6 (4.3) | 17.5 (11) | 9.7 (7.2) | 3.4 (3.7) | 13 (10.2) | 6.6 (6.4) | 2.4 (3.6) | 0.2 (1.6) |
| 39 | Lanao Del Norte | 19658 | 24.3 (0.5) | 270.5 (112.4) | 86.8 (2.3) | 15.6 (15.4) | 9.3 (9.8) | 2.5 (4) | 9 (12.6) | 4.7 (7) | 0.7 (1.6) | 1.6 (1.8) |
| 40 | Lanao Del Sur | 6598 | 22.1 (0.5) | 347.2 (126.8) | 88.2 (2.5) | 16.1 (22.8) | 9.6 (16.2) | 2.3 (5.1) | 10 (19.7) | 5.8 (12.7) | 1.1 (2.6) | 1.6 (1.8) |
| 41 | Leyte | 33947 | 25.9 (0.8) | 237.9 (135.8) | 84.5 (3.1) | 17.5 (15) | 9.5 (8.9) | 2.9 (4) | 14.3 (14) | 6.7 (7.2) | 1.7 (3.3) | 1.8 (1.7) |
| 42 | Maguindanao | 11711 | 26.4 (0.8) | 221.2 (92.7) | 80.1 (5.1) | 16.5 (24.6) | 10.1 (17) | 2.1 (4.3) | 12.3 (21.5) | 6.9 (12.9) | 1.7 (3.9) | 1.5 (2) |
| 43 | Marinduque | 2583 | 26.2 (1) | 187.7 (117.6) | 81.6 (3.4) | 17.9 (12.4) | 10.8 (9.1) | 3.5 (5.2) | 14.5 (12.1) | 7.2 (7.5) | 2.5 (3.9) | 0.5 (1.8) |
| **N** | **Province** | **Total dengue** | **Mean monthly temperature (SD)** | **Mean monthly total precipitation (SD)** | **Mean monthly relative humidity (SD)** | **Mean annual hw.2.95 (SD)** | **Mean annual hw.2.97 (SD)** | **Mean annual hw.2.99 (SD)** | **Mean annual hw.4.95 (SD)** | **Mean annual hw.4.97 (SD)** | **Mean annual hw.4.99 (SD)** | **Monthly mean scpdsi (SD)** |
| 44 | Masbate | 2713 | 26.8 (0.8) | 201.6 (130.1) | 81.9 (2.7) | 17.5 (13.5) | 10.4 (10.6) | 3.4 (4.5) | 12.5 (12.2) | 7.5 (9.8) | 2.2 (3.9) | 1.9 (1.5) |
| 45 | Metropolitan Manila | 266489 | 26.8 (1.2) | 190.1 (167.8) | 78.8 (6.7) | 17.9 (12.1) | 9.7 (7.9) | 2.9 (3.7) | 13.5 (11.8) | 7.5 (7.2) | 2.4 (3.6) | 0.1 (1.7) |
| 46 | Mindoro Occidental | 6640 | 24.6 (0.9) | 287.8 (241.6) | 83.5 (5.3) | 17.8 (14) | 10.3 (11.2) | 3.2 (5.7) | 13.8 (13.4) | 7.6 (10.6) | 3 (5.5) | 0.3 (1.7) |
| 47 | Mindoro Oriental | 11804 | 24.6 (1.1) | 276.8 (151.4) | 85.3 (3.4) | 17.5 (13.1) | 10.1 (10.2) | 3.2 (5.6) | 13.5 (12.2) | 7.3 (9.5) | 3.2 (5.6) | 0.4 (1.7) |
| 48 | Misamis Occidental | 14130 | 24.5 (0.5) | 241.9 (104.3) | 86.3 (2.3) | 15.5 (12.9) | 8.7 (8.6) | 2.8 (3.2) | 9.2 (9.2) | 4.8 (6.9) | 0.4 (1.2) | 1.7 (1.9) |
| 49 | Misamis Oriental | 48658 | 24 (0.6) | 245.8 (128.8) | 84.3 (2.9) | 15.2 (15.4) | 8.5 (9.5) | 2 (3.4) | 11 (13.6) | 5.3 (8) | 0.7 (1.6) | 1.5 (1.9) |
| 50 | Mountain Province | 5117 | 20.7 (1.6) | 267.8 (154.8) | 85.8 (3) | 15.9 (9.4) | 10 (8.3) | 2.8 (4.1) | 10.1 (8.2) | 5.7 (5.8) | 2.3 (4.2) | -0.1 (1.5) |
| 51 | Negros Occidental | 66540 | 25.5 (0.8) | 261.6 (124) | 84.6 (4.5) | 17.7 (19) | 10.9 (14.9) | 3.3 (6.3) | 16.1 (18.8) | 9.5 (14.4) | 2.8 (6.3) | 2.1 (1.3) |
| 52 | Negros Oriental | 33557 | 25 (0.7) | 210.8 (114.9) | 83.9 (4.8) | 17.5 (18.9) | 10.6 (15.2) | 3.1 (6.5) | 15.7 (18.6) | 9.1 (14.3) | 2.3 (6.3) | 1.9 (1.4) |
| 53 | North Cotabato | 32465 | 25.1 (0.8) | 230.7 (96.4) | 80.6 (5.4) | 16.6 (24.2) | 10.1 (17.9) | 2.5 (5.2) | 12 (22.2) | 7.1 (14.9) | 1.5 (3.7) | 1.5 (2) |
| 54 | Northern Samar | 8582 | 26.3 (0.8) | 219.5 (118.5) | 84.2 (3.2) | 17.5 (12.6) | 9.7 (8.3) | 3.1 (4.7) | 11.5 (10.7) | 6.2 (7.5) | 1.1 (3.6) | 1.1 (1.7) |
| 55 | Nueva Ecija | 48321 | 25.3 (1.3) | 195.3 (154) | 80.6 (6.5) | 17.1 (11.7) | 10.3 (9.2) | 2.8 (3.5) | 14.5 (11.4) | 7.2 (8.3) | 1.5 (2.5) | -0.5 (1.4) |
| 56 | Nueva Vizcaya | 19865 | 21.9 (1.6) | 222.3 (120.3) | 84.6 (3.2) | 15.7 (13) | 9.5 (10) | 2.4 (3.6) | 11.2 (11.7) | 6.4 (7.5) | 0.9 (2.1) | -0.7 (1.5) |
| 57 | Palawan | 23676 | 26.2 (0.7) | 209.6 (113.6) | 83 (3.5) | 17.7 (20.5) | 11.1 (14.8) | 2.6 (4.4) | 13.5 (19.1) | 8.8 (13) | 1.1 (2.5) | 0.4 (0.4) |
| 58 | Pampanga | 68005 | 26.1 (1.3) | 167.8 (139.4) | 78.9 (7.3) | 18.3 (11.4) | 10.5 (8.9) | 3.2 (3.9) | 15.9 (11.5) | 7.4 (8.3) | 2.5 (3.7) | 0 (1.5) |
| 59 | Pangasinan | 61696 | 26.2 (1.1) | 194.6 (176.6) | 78 (7.4) | 17.8 (12.4) | 10.3 (10.4) | 2.7 (4.8) | 14.6 (12.3) | 7.9 (10.5) | 2.2 (3.8) | -0.5 (1.2) |
| 60 | Quezon | 40043 | 25.8 (1.1) | 221.6 (122.7) | 83.5 (3.2) | 17.5 (12.3) | 10.8 (8.6) | 3.3 (4.4) | 13.9 (10.8) | 7.7 (7.7) | 2.6 (4) | 0.3 (1.6) |
| 61 | Quirino | 9633 | 23.1 (1.8) | 237.1 (131) | 85.7 (2.9) | 15.5 (12.3) | 8.3 (8.2) | 2 (3.5) | 8.3 (10.1) | 3.4 (4.8) | 0.9 (2.1) | -0.8 (1.5) |
| 62 | Rizal | 54135 | 25.4 (1.2) | 220.3 (173.1) | 82.6 (4.9) | 17.5 (11) | 9.7 (7.2) | 3.4 (3.7) | 13 (10.2) | 6.6 (6.4) | 2.4 (3.6) | 0 (1.7) |
| 63 | Romblon | 2918 | 26.3 (0.8) | 237.5 (141.4) | 82.2 (3.2) | 17.6 (14) | 10.1 (10.8) | 2.8 (3.9) | 12.5 (13.6) | 7.1 (10.5) | 1.3 (2.2) | 1.6 (1.6) |
| 64 | Samar | 15096 | 25.7 (0.8) | 215 (120.8) | 84.7 (3.3) | 16.9 (12.3) | 10.2 (8.6) | 3.1 (4.4) | 11.5 (10.8) | 5.8 (7.6) | 0.8 (2.7) | 1.3 (1.7) |
| 65 | Saranggani | 12426 | 24.3 (0.5) | 245.7 (97.1) | 86.2 (3.6) | 16.1 (23.3) | 10.2 (17.4) | 3.2 (6.3) | 12.1 (21.5) | 7.3 (13.6) | 1.3 (3.1) | 1 (2.1) |
| **N** | **Province** | **Total dengue** | **Mean monthly temperature (SD)** | **Mean monthly total precipitation (SD)** | **Mean monthly relative humidity (SD)** | **Mean annual hw.2.95 (SD)** | **Mean annual hw.2.97 (SD)** | **Mean annual hw.2.99 (SD)** | **Mean annual hw.4.95 (SD)** | **Mean annual hw.4.97 (SD)** | **Mean annual hw.4.99 (SD)** | **Monthly mean scpdsi (SD)** |
| 66 | Siquijor | 3421 | 26.5 (0.6) | 171.3 (92.1) | 82.3 (1.4) | 16.9 (18.5) | 9.8 (12.4) | 3.5 (6.3) | 14.4 (16.8) | 7.6 (11) | 2.4 (5.4) | 2.1 (1.6) |
| 67 | Sorsogon | 4998 | 26.3 (0.8) | 221.2 (120.6) | 83.9 (2.9) | 17.4 (13.6) | 10.2 (10.1) | 3.3 (4.5) | 11.9 (12.2) | 6.7 (8.4) | 2.2 (3.5) | 1.1 (1.7) |
| 68 | South Cotabato | 50820 | 23.9 (0.7) | 228.7 (90.8) | 84.7 (5.3) | 16.2 (23.6) | 10.1 (16.8) | 2.3 (4.6) | 11.6 (20.2) | 7.3 (13.9) | 1.7 (3.9) | 1.4 (2) |
| 69 | Southern Leyte | 4391 | 25.6 (0.8) | 243.4 (125.5) | 85.4 (3) | 16.3 (12.4) | 8.9 (6.9) | 3.4 (3.2) | 10.9 (9) | 6.4 (4.7) | 1.9 (2.2) | 1.6 (1.7) |
| 70 | Sultan Kudarat | 9190 | 24.4 (0.6) | 264.8 (109.6) | 83.5 (4.3) | 15.8 (24) | 9.9 (17.1) | 2.3 (4.6) | 11.9 (20.5) | 7.2 (13.5) | 1.7 (3.9) | 1.3 (2.1) |
| 71 | Sulu | 938 | 26.8 (0.4) | 120.2 (64.8) | 82.6 (1.1) | 13.1 (12.2) | 7.3 (7.5) | 2 (4.2) | 5.6 (8.6) | 3.3 (7.3) | 0.8 (2.7) | 0.1 (2.1) |
| 72 | Surigao Del Norte | 12746 | 25.8 (0.8) | 236.2 (175.5) | 83.9 (2.7) | 15.5 (12.7) | 9.3 (9.3) | 2.9 (4.4) | 10.1 (10) | 6.8 (8.7) | 1.8 (2.6) | 0.1 (1.6) |
| 73 | Surigao Del Sur | 15818 | 25.3 (0.7) | 275.7 (145.6) | 86.5 (2.2) | 15.2 (13.6) | 9.3 (9) | 2.7 (4) | 10.7 (10.3) | 6.2 (7.5) | 1.9 (2.8) | 0.8 (1.8) |
| 74 | Tarlac | 41559 | 25.6 (1.3) | 173.9 (145) | 78.4 (7.9) | 17.6 (11.4) | 9.5 (9.6) | 3.1 (4.2) | 15.5 (12.2) | 7 (8.1) | 1.7 (3) | -0.4 (1.3) |
| 75 | Tawi-tawi | 978 | 27.2 (0.4) | 142.4 (69.1) | 81.6 (1.3) | 13.7 (12.2) | 6.7 (6.4) | 2.1 (4.4) | 7.1 (8.9) | 2.1 (4.3) | 1.2 (2.9) | 0.4 (2.2) |
| 76 | Zambales | 15019 | 25.1 (1) | 239.9 (218.7) | 80.7 (7.5) | 18.2 (11.3) | 10 (9.3) | 3.4 (4.2) | 15.5 (11.2) | 7.5 (8.4) | 2.5 (3.7) | -0.3 (1.2) |
| 77 | Zamboanga Del Norte | 16515 | 25.3 (0.5) | 256.1 (116.1) | 85.7 (3.2) | 15.7 (15.1) | 9.8 (10) | 2.8 (4.2) | 10 (11.4) | 5.6 (7.4) | 1 (2.3) | 1.3 (1.8) |
| 78 | Zamboanga Del Sur | 45079 | 25.4 (0.6) | 240.7 (110.7) | 85 (4.1) | 14.7 (15.9) | 8.3 (9) | 2.8 (4.4) | 7.8 (10.3) | 4.1 (6.3) | 1.8 (3.2) | 1.2 (1.8) |
| 79 | Zamboanga Sibugay | 11357 | 26.1 (0.5) | 242.1 (105.9) | 84.7 (3.9) | 14.1 (17.2) | 8.8 (12) | 2.4 (4.8) | 7.4 (12.1) | 4.7 (7.2) | 0.7 (2.4) | 1.2 (1.9) |

Note: SD: standard deviation; hw.2.95: heatwave with 95^th^ percentile for 2 consecutive days; hw.2.97: heatwave with 97^th^ percentile for 2 consecutive days; hw.2.99: heatwave with 99^th^ percentile for 2 consecutive days; hw.4.95: heatwave with 95^th^ percentile for 4 consecutive days; hw.4.97: heatwave with 97^th^ percentile for 4 consecutive days; hw.4.99: heatwave with 99^th^ percentile for 4 consecutive days; scPDSI: self-calibrated Palmer Drought Severity Index.6. Singapore

| **N** | **Province** | **Total dengue** | **Mean monthly temperature (SD)** | **Mean monthly total precipitation (SD)** | **Mean monthly relative humidity (SD)** | **Mean annual hw.2.95 (SD)** | **Mean annual hw.2.97 (SD)** | **Mean annual hw.2.99 (SD)** | **Mean annual hw.4.95 (SD)** | **Mean annual hw.4.97 (SD)** | **Mean annual hw.4.99 (SD)** | **Monthly mean scpdsi (SD)** |
| --- | --- | --- | --- | --- | --- | --- | --- | --- | --- | --- | --- | --- |
| 1 | Singapore | 91463 | 26.9 (0.6) | 206.5 (89.8) | 83.5 (2.3) | 14.6 (11.1) | 8.8 (7.5) | 1.9 (1.9) | 8.9 (8) | 3 (4.1) | 0 (0) | 0.8 (2) |

Note: SD: standard deviation; hw.2.95: heatwave with 95^th^ percentile for 2 consecutive days; hw.2.97: heatwave with 97^th^ percentile for 2 consecutive days; hw.2.99: heatwave with 99^th^ percentile for 2 consecutive days; hw.4.95: heatwave with 95^th^ percentile for 4 consecutive days; hw.4.97: heatwave with 97^th^ percentile for 4 consecutive days; hw.4.99: heatwave with 99^th^ percentile for 4 consecutive days; scPDSI: self-calibrated Palmer Drought Severity Index.

7. Thailand

| **N** | **Province** | **Total dengue** | **Mean monthly temperature (SD)** | **Mean monthly total precipitation (SD)** | **Mean monthly relative humidity (SD)** | **Mean annual hw.2.95 (SD)** | **Mean annual hw.2.97 (SD)** | **Mean annual hw.2.99 (SD)** | **Mean annual hw.4.95 (SD)** | **Mean annual hw.4.97 (SD)** | **Mean annual hw.4.99 (SD)** | **Monthly mean scpdsi (SD)** |
| --- | --- | --- | --- | --- | --- | --- | --- | --- | --- | --- | --- | --- |
| 1 | Amnat Charoen | 5457 | 26.2 (2.2) | 125.4 (128.6) | 71.1 (10.2) | 16.8 (12.6) | 9.9 (9.7) | 3.2 (6.2) | 12.1 (11.5) | 6.8 (9.5) | 1.6 (5.6) | -0.3 (2.3) |
| 2 | Ang Thong | 6137 | 28.1 (1.7) | 105.6 (93.2) | 66.9 (9.8) | 17.2 (13.4) | 9.9 (10) | 3.2 (5.8) | 12.2 (12.3) | 7.1 (8.7) | 1.6 (5.8) | 0.3 (2.1) |
| 3 | Bangkok | 177381 | 27.8 (1.4) | 113.7 (92) | 72.8 (7.3) | 17.2 (12.7) | 10.3 (10.5) | 3.4 (5.2) | 13.4 (11.7) | 7.9 (10.6) | 1.8 (4.4) | 0.3 (1.8) |
| 4 | Buriram | 30599 | 26.6 (2) | 105.4 (92.3) | 68.9 (10.1) | 16.4 (14.9) | 9.8 (11.8) | 3.4 (6.4) | 12.5 (13.4) | 6.8 (10.8) | 1.7 (5.4) | 0.1 (1.9) |
| 5 | Chachoengsao | 16794 | 27.1 (1.2) | 134.4 (104.7) | 74.3 (9.2) | 17.8 (14.2) | 10.4 (10.6) | 3.6 (6.8) | 13.8 (13.2) | 7.3 (9.8) | 2.5 (5.8) | 0.2 (1.8) |
| 6 | Chainat | 5644 | 27.9 (2) | 103.6 (92.6) | 66.7 (10.3) | 17.1 (15.2) | 10.3 (12.9) | 3.7 (7.2) | 12.7 (15.2) | 6.8 (11.6) | 2.4 (7) | 0.6 (2) |
| 7 | Chaiyaphum | 19480 | 25.1 (2) | 120.2 (106.7) | 72.3 (10.4) | 17.7 (14.9) | 10.3 (13) | 3.5 (6.7) | 12.5 (14.1) | 7 (11.7) | 2.1 (6) | -0.3 (2) |
| 8 | Chanthaburi | 18594 | 25.8 (0.9) | 190.1 (142) | 80.9 (9.5) | 16.6 (18.2) | 10.2 (14.3) | 2.9 (6.5) | 12.6 (17) | 6.8 (11.8) | 1.4 (4.7) | 0.1 (1.8) |
| 9 | Chiang Mai | 53507 | 22.7 (2.1) | 137.9 (125.2) | 73.5 (14.7) | 17.5 (15.7) | 10.7 (13.5) | 3.7 (7.9) | 14.5 (15.7) | 8.1 (13.2) | 2.7 (6.9) | -0.5 (2) |
| 10 | Chiang Rai | 36481 | 23.9 (2.5) | 147.1 (132.8) | 73 (12.9) | 18.2 (15.7) | 10.7 (13.5) | 3.7 (8.4) | 14.7 (15.3) | 8 (12.9) | 2.5 (6.2) | -0.2 (2.1) |
| 11 | Chonburi | 28671 | 27 (1.1) | 125.3 (92.6) | 75.5 (8.3) | 17.8 (14.1) | 10.3 (11) | 3.4 (6.5) | 13.8 (12.8) | 7.6 (10.8) | 2 (5.4) | 0.5 (1.8) |
| 12 | Chumphon | 10498 | 25.8 (0.9) | 173.4 (112.9) | 83.1 (5.7) | 17.6 (16.9) | 10.4 (13.1) | 3.6 (8.1) | 14.7 (15.7) | 8.7 (12.3) | 2.9 (7.9) | 0 (1.9) |
| 13 | Kalasin | 12914 | 26.1 (2.3) | 113.4 (111.6) | 69.4 (10.4) | 16.7 (12.5) | 10.3 (10.8) | 3.4 (5.8) | 11.5 (11.4) | 6.4 (10.3) | 1.6 (4.7) | -0.1 (2.4) |
| 14 | Kampaeng Phet | 12734 | 26.1 (2) | 134.4 (120.5) | 73 (11.1) | 17.3 (14.2) | 10.5 (11.7) | 3.6 (7.9) | 14.2 (14.1) | 9.1 (11.8) | 2.3 (7.1) | 0 (2.3) |
| 15 | Kanchanaburi | 12654 | 24.9 (1.5) | 141.2 (121.6) | 75.7 (11.1) | 16.2 (15.6) | 10 (12.8) | 3.3 (6.5) | 11.6 (14.3) | 7.6 (12.1) | 2.2 (5.8) | -0.1 (1.8) |
| 16 | Khon Kaen | 25660 | 26.3 (2.3) | 103 (97.1) | 68.3 (10.1) | 17.7 (13.9) | 10.2 (11.7) | 3.5 (5.9) | 13.1 (12.9) | 6.7 (11) | 1.4 (4) | -0.5 (2.2) |
| **N** | **Province** | **Total dengue** | **Mean monthly temperature (SD)** | **Mean monthly total precipitation (SD)** | **Mean monthly relative humidity (SD)** | **Mean annual hw.2.95 (SD)** | **Mean annual hw.2.97 (SD)** | **Mean annual hw.2.99 (SD)** | **Mean annual hw.4.95 (SD)** | **Mean annual hw.4.97 (SD)** | **Mean annual hw.4.99 (SD)** | **Monthly mean scpdsi (SD)** |
| 17 | Krabi | 16041 | 26.3 (1) | 189.8 (110) | 83 (7.9) | 17.2 (20.7) | 10.4 (15.7) | 3.3 (7.8) | 15 (18.9) | 8.6 (14.2) | 2.1 (7.1) | 0.3 (2) |
| 18 | Lampang | 13835 | 24.9 (2.2) | 125 (115.2) | 71.9 (14.6) | 18.1 (15.2) | 10.6 (12.5) | 3.6 (7.6) | 14.5 (14.5) | 8.6 (12.5) | 2.5 (6.4) | -0.4 (2) |
| 19 | Lamphun | 6667 | 24.6 (2.2) | 115 (106) | 70.4 (14.5) | 17.7 (15.6) | 10.6 (12.7) | 3.6 (7.9) | 14.5 (15) | 7.9 (12.3) | 2.7 (7.2) | -0.1 (2.1) |
| 20 | Loei | 12136 | 24.4 (2.2) | 120.1 (105.1) | 72.6 (10.2) | 17.1 (15.4) | 10.7 (13) | 3.4 (6.9) | 12.8 (13.6) | 7.4 (11.7) | 2.6 (6.6) | 0.2 (2.1) |
| 21 | Lopburi | 17364 | 27.4 (1.8) | 108.3 (99.7) | 68.3 (10.8) | 17.3 (14.8) | 9.9 (11.7) | 3.3 (6.5) | 13.2 (14.9) | 7.2 (11.1) | 1.9 (6.2) | 0.1 (2) |
| 22 | Mae Hong Son | 10469 | 22.7 (2.1) | 131.1 (125.7) | 74 (16.3) | 17.5 (15.2) | 10.1 (14.3) | 3.7 (8.9) | 14.2 (15.3) | 8.3 (14.5) | 3.2 (8.3) | -0.8 (2) |
| 23 | Maha Sarakham | 14127 | 26.7 (2.2) | 106.7 (102.1) | 67.3 (10.1) | 17.2 (13.5) | 10 (10.4) | 3.5 (6.2) | 12.1 (12.2) | 6.4 (10) | 1.5 (5.2) | -0.7 (2.3) |
| 24 | Mukdahan | 6712 | 25.5 (2.4) | 126.4 (128.5) | 72.2 (10.1) | 17.1 (13.5) | 9.8 (10.1) | 3.5 (5.9) | 11.8 (12.4) | 6.6 (9.2) | 1.8 (4.6) | -0.1 (2.3) |
| 25 | Nakhon Nayok | 4350 | 26.3 (1.4) | 141 (117.7) | 74.9 (9.2) | 17.5 (15.2) | 10.2 (12.1) | 3.2 (6.8) | 13.8 (14.7) | 7.6 (11.5) | 1.7 (6.4) | 0.1 (1.9) |
| 26 | Nakhon Pathom | 27129 | 27.9 (1.5) | 108.2 (88.7) | 69.8 (8.3) | 17.5 (13.4) | 10.5 (10.8) | 3.3 (6.4) | 14.1 (12.8) | 7.9 (10.3) | 2 (6.3) | 0.3 (1.8) |
| 27 | Nakhon Phanom | 9355 | 25.5 (2.5) | 154.3 (162.6) | 72.6 (10.7) | 17.1 (13.5) | 10.2 (10.6) | 3.2 (5.1) | 12.2 (12.6) | 6.2 (9.6) | 2.1 (4.7) | -0.1 (2.4) |
| 28 | Nakhon Ratchasima | 61389 | 26 (1.9) | 110.3 (92.4) | 70.5 (9.7) | 17.1 (15.6) | 10.4 (13.4) | 3.4 (7) | 12.3 (14.1) | 6.8 (12) | 1.8 (5.3) | 0.2 (1.9) |
| 29 | Nakhon Sawan | 29019 | 27.3 (2) | 115.9 (103.5) | 69 (10.9) | 16.8 (15.1) | 10.3 (13) | 3.6 (7.2) | 12.4 (14.9) | 7.3 (11.9) | 2.4 (7) | 0 (2) |
| 30 | Nakhon Si Thammarat | 45215 | 25.9 (1) | 171.9 (111.2) | 82.6 (5.3) | 16.9 (18.1) | 10.4 (13.3) | 3.5 (8.5) | 13.8 (16.1) | 7.8 (12.3) | 2.6 (8.2) | 0.2 (2) |
| 31 | Nan | 5159 | 23.7 (2.1) | 150.5 (141.6) | 74.5 (13.1) | 17.7 (16) | 10.5 (13.3) | 3.5 (7.3) | 13.3 (15) | 7.2 (12.7) | 2.5 (6.2) | 0.8 (2.3) |
| 32 | Narathiwat | 19012 | 25.4 (1.1) | 206.8 (124.3) | 85 (4.4) | 14.6 (18.3) | 9.1 (13.7) | 2.9 (7.2) | 11.1 (15.9) | 6.5 (12.3) | 1.9 (7.2) | -0.8 (1.9) |
| 33 | Nong Bua Lamphu | 4458 | 25.9 (2.5) | 113.7 (107.6) | 69.9 (10.4) | 17 (12.3) | 10.3 (10.7) | 3.5 (5.6) | 11.9 (12.4) | 5.7 (10.3) | 1.2 (3.6) | -0.3 (2.5) |
| 34 | Nong Khai | 7166 | 25.5 (2.4) | 170.3 (178) | 72.4 (11.5) | 16.6 (12) | 10.2 (10) | 3.2 (4.4) | 10.8 (11.8) | 6.2 (9.2) | 1.4 (2.9) | -0.1 (2.4) |
| 35 | Nonthaburi | 23948 | 27.9 (1.5) | 109 (91.2) | 70.9 (8) | 17.3 (12.5) | 10.2 (10.4) | 3.6 (5.7) | 13.4 (11.9) | 8.3 (10.6) | 1.9 (5.2) | 0.3 (1.8) |
| 36 | Pathum Thani | 14281 | 27.7 (1.4) | 116.7 (96.4) | 71.9 (8.2) | 17.3 (13.1) | 10.1 (10.4) | 3.1 (5.4) | 13.3 (12.4) | 7.3 (9.8) | 1.5 (5.3) | 0.2 (1.9) |
| 37 | Pattani | 16857 | 26.6 (1) | 169.1 (114.6) | 81.3 (5.2) | 13.9 (16.8) | 9.2 (12.3) | 3.2 (7.7) | 10.6 (13.5) | 6.3 (10.8) | 2.1 (7.6) | -0.7 (1.8) |
| 38 | Phachinburi | 13966 | 26.2 (1.4) | 139.1 (118.7) | 75.1 (10.2) | 17.4 (15.2) | 10.1 (12.2) | 3.4 (6.7) | 13.7 (14.5) | 7.5 (11.1) | 2.1 (5.6) | 0.4 (1.9) |
| 39 | Phangnga | 8053 | 25.9 (0.9) | 220.5 (129.4) | 83.5 (7.3) | 17 (20.4) | 10.4 (16.2) | 3 (6.9) | 16 (19.9) | 8 (15) | 1.8 (5.3) | 0.3 (2) |
| 40 | Phatthalung | 13743 | 26.1 (1) | 172.9 (119.4) | 83.7 (4.3) | 15.3 (18) | 9.2 (14.2) | 3.4 (8.5) | 10.9 (15.2) | 5.8 (11.2) | 2.4 (8.3) | -0.2 (1.7) |
| 41 | Phayao | 5919 | 24.5 (2.3) | 135.7 (124.5) | 72 (13.3) | 17.7 (16.5) | 10.9 (13.8) | 3.5 (7.7) | 13.8 (15.2) | 8.3 (13) | 2.5 (6.2) | 0.1 (2.2) |
| 42 | Phetchabun | 20984 | 25.3 (1.8) | 140.9 (122.9) | 73.2 (11.2) | 17.5 (14.8) | 10.4 (12.2) | 3.5 (7.3) | 12.6 (14.1) | 6.6 (11.6) | 2.1 (6.4) | -0.1 (2) |
| 43 | Phetchaburi | 14397 | 25.5 (1.3) | 123.5 (91.7) | 75.9 (8.3) | 17 (16.7) | 10.2 (13) | 3.2 (7.7) | 14.3 (15.3) | 8.4 (12) | 1.7 (6.7) | 0.7 (1.7) |
| **N** | **Province** | **Total dengue** | **Mean monthly temperature (SD)** | **Mean monthly total precipitation (SD)** | **Mean monthly relative humidity (SD)** | **Mean annual hw.2.95 (SD)** | **Mean annual hw.2.97 (SD)** | **Mean annual hw.2.99 (SD)** | **Mean annual hw.4.95 (SD)** | **Mean annual hw.4.97 (SD)** | **Mean annual hw.4.99 (SD)** | **Monthly mean scpdsi (SD)** |
| 44 | Phichit | 13022 | 27.4 (2) | 133.9 (126.8) | 70.3 (11.6) | 17.3 (13.5) | 10.6 (11.1) | 3.4 (7.3) | 13.6 (13.3) | 7.8 (11) | 2.6 (7.2) | -0.1 (2.2) |
| 45 | Phitsanulok | 15525 | 25.6 (2) | 151 (134) | 73.7 (11.5) | 17.6 (13.2) | 10.8 (11.2) | 3.5 (7.5) | 13.4 (13.1) | 7.8 (10.9) | 2.6 (7.2) | 0.1 (2.2) |
| 46 | Phra Nakhon Si Ayudhya | 12580 | 27.9 (1.5) | 110.2 (94.3) | 69.3 (9.2) | 17.2 (13.4) | 9.9 (10) | 3.2 (5.8) | 12.2 (12.3) | 7.1 (8.7) | 1.6 (5.8) | 0.2 (1.9) |
| 47 | Phrae | 8024 | 25.3 (2.1) | 135 (128.4) | 73.4 (14) | 17.5 (14.9) | 10.6 (12.4) | 3.5 (7.5) | 13.2 (13.8) | 7.6 (12.2) | 2.6 (6.6) | 0 (2.2) |
| 48 | Phuket | 10228 | 27.2 (0.6) | 186.7 (122.2) | 79.4 (3.5) | 16.7 (19.8) | 10.5 (16.3) | 2.7 (6.3) | 14.6 (19.1) | 8.3 (15.7) | 1.4 (4.4) | 0.3 (2) |
| 49 | Prachuap Khilikhan | 9855 | 25.7 (1.1) | 137.7 (94.3) | 79 (7.3) | 17.7 (17.9) | 10.6 (14.6) | 3.3 (7) | 14.5 (17.5) | 8.6 (12.7) | 1.5 (5.5) | 0.6 (1.8) |
| 50 | Ranong | 3234 | 25.4 (0.9) | 215.3 (139.3) | 84.2 (7.4) | 17.6 (19.1) | 10.4 (13.5) | 3.4 (7.2) | 14.6 (18) | 8.1 (11.9) | 2.3 (6.9) | 0 (1.9) |
| 51 | Ratchaburi | 27928 | 26.1 (1.5) | 113.8 (88.2) | 74.2 (8.8) | 16.8 (15.3) | 9.9 (11.6) | 3 (6.2) | 13.8 (14.5) | 7.3 (9.9) | 1.8 (5.8) | 0.2 (1.8) |
| 52 | Rayong | 29826 | 26.7 (0.9) | 150.3 (110.3) | 78.2 (8.3) | 17 (15.7) | 10.1 (11.2) | 3.5 (7) | 13.4 (13.6) | 6.5 (10.5) | 2.3 (5.9) | 0.6 (1.8) |
| 53 | Roi Et | 28533 | 26.6 (2.2) | 113.8 (111.7) | 68.4 (10.1) | 17.3 (13.2) | 10.2 (10) | 3.4 (6.1) | 12.4 (12) | 7.3 (10.2) | 1.5 (5.4) | -0.1 (2.2) |
| 54 | Sa Kaeo | 11469 | 26.5 (1.4) | 125.1 (101.1) | 74 (10.6) | 16.9 (17) | 10.2 (13.6) | 3.2 (6.4) | 12.4 (15.6) | 7.2 (12.5) | 1.6 (4.9) | 0.7 (1.9) |
| 55 | Sakon Nakhon | 9040 | 25.7 (2.5) | 140.8 (143.3) | 70.7 (10.6) | 17.4 (13) | 10.3 (9.9) | 3.4 (4.8) | 11.7 (12.3) | 6 (9.9) | 1.5 (3.3) | -0.1 (2.5) |
| 56 | Samut Prakarn | 28801 | 27.9 (1.3) | 107.2 (88.1) | 72.7 (7) | 17.2 (12.7) | 10.3 (10.5) | 3.4 (5.2) | 13.4 (11.7) | 7.9 (10.6) | 1.8 (4.4) | 0.2 (1.8) |
| 57 | Samut Sakhon | 16833 | 27.9 (1.4) | 103.1 (86.2) | 72.7 (6.5) | 17.2 (12.7) | 10.3 (10.5) | 3.4 (5.2) | 13.4 (11.7) | 7.9 (10.6) | 1.8 (4.4) | 0.4 (1.8) |
| 58 | Samut Songkham | 4116 | 27.8 (1.5) | 90.8 (78.2) | 72 (6.5) | 17.1 (14.4) | 10.4 (11.2) | 3.3 (6.7) | 14.1 (14.3) | 7.3 (10.1) | 1.8 (6) | 0.7 (1.7) |
| 59 | Saraburi | 10531 | 26.8 (1.5) | 118.7 (99.6) | 71.1 (10) | 16.9 (14.5) | 10 (10.7) | 3.1 (6.7) | 13.6 (14.4) | 7.4 (10) | 1.8 (6.7) | 0.2 (2) |
| 60 | Satun | 4734 | 26.3 (0.8) | 188.4 (110.2) | 83.2 (6.8) | 15.8 (19.2) | 9.2 (14.9) | 2.7 (8.7) | 11.6 (16.8) | 5.7 (12) | 1.6 (6.9) | -0.1 (2) |
| 61 | Si Saket | 31857 | 26.4 (1.9) | 120.2 (107.9) | 71.3 (10.3) | 16.2 (13) | 9.7 (9.8) | 3.2 (5.1) | 10.7 (11.2) | 6.4 (8.9) | 1.8 (4.8) | 0.1 (2.1) |
| 62 | Singburi | 1572 | 28.1 (1.8) | 102.5 (94.5) | 66.2 (10.2) | 17 (13.9) | 10.2 (10.7) | 3.4 (6.2) | 12.8 (13.2) | 6.9 (9.2) | 1.7 (6.3) | 0.4 (2.1) |
| 63 | Songkhla | 45751 | 26.2 (1) | 168.5 (110.4) | 82.2 (6.1) | 15.6 (20.1) | 9.1 (16.2) | 3.1 (9) | 11.1 (17.1) | 6.9 (14.8) | 1.4 (6) | -0.5 (1.7) |
| 64 | Sukhothai | 8095 | 26.9 (2.3) | 123.4 (114.9) | 70.5 (13.1) | 17.4 (13.8) | 10.6 (11.7) | 3.3 (7.3) | 14.1 (13.1) | 8.6 (11.7) | 2.5 (7.1) | -0.2 (2.2) |
| 65 | Suphanburi | 13247 | 27.5 (1.7) | 103.4 (87.7) | 68.2 (9.6) | 17.4 (14.9) | 10.2 (11.7) | 3.6 (6.8) | 13.3 (14.7) | 7.4 (10.9) | 2.2 (6.6) | 0.3 (1.9) |
| 66 | Surat Thani | 17338 | 25.8 (1.1) | 175 (109.9) | 82.7 (7.1) | 17.6 (19.9) | 10.8 (14.5) | 3.5 (8) | 15.5 (18.1) | 8.6 (12.6) | 2.6 (7.7) | 0.4 (2) |
| 67 | Surin | 39121 | 26.6 (2) | 113.3 (103.7) | 69.9 (10.3) | 16.4 (14.3) | 9.7 (9.9) | 3.3 (5.4) | 12.3 (12.7) | 6.8 (9.6) | 2.1 (5.2) | -0.4 (2) |
| 68 | Tak | 17540 | 23.6 (1.7) | 153.3 (141.9) | 75.4 (13.9) | 17.4 (16.1) | 10 (12.2) | 3.6 (7.8) | 13.1 (15.4) | 7.5 (12.2) | 2.6 (7.1) | -0.4 (2) |
| 69 | Trad | 6451 | 25.8 (0.8) | 243 (188.1) | 82.9 (9.3) | 16.4 (19.7) | 9.4 (14.8) | 2.9 (7) | 10.8 (16) | 6.3 (12.7) | 1.6 (5) | 0.1 (1.9) |
| **N** | **Province** | **Total dengue** | **Mean monthly temperature (SD)** | **Mean monthly total precipitation (SD)** | **Mean monthly relative humidity (SD)** | **Mean annual hw.2.95 (SD)** | **Mean annual hw.2.97 (SD)** | **Mean annual hw.2.99 (SD)** | **Mean annual hw.4.95 (SD)** | **Mean annual hw.4.97 (SD)** | **Mean annual hw.4.99 (SD)** | **Monthly mean scpdsi (SD)** |
| 70 | Trang | 9982 | 26.1 (0.9) | 183.4 (110.5) | 83.1 (6.2) | 15.4 (18.6) | 9.5 (15) | 3.3 (8.3) | 11.2 (17) | 6.4 (11.6) | 2.2 (8.1) | 0.2 (2) |
| 71 | Ubon Ratchathani | 35601 | 26.3 (1.9) | 136.5 (129.8) | 72.3 (11.1) | 16 (13.4) | 9.8 (10.1) | 3 (5.6) | 10.4 (11.6) | 6.5 (9.5) | 1.6 (4.7) | -0.1 (2.2) |
| 72 | Udon Thani | 10449 | 26 (2.4) | 128.9 (126.9) | 69.5 (11) | 16.9 (12.6) | 10.6 (10.6) | 3.4 (4.9) | 11.1 (11.7) | 6.5 (10.4) | 1.4 (3.7) | -0.3 (2.4) |
| 73 | Uthai Thani | 6834 | 25.2 (1.8) | 122 (100.7) | 73.7 (10.3) | 16.5 (15.8) | 10.1 (12.7) | 3.1 (7.7) | 13.1 (16) | 7.3 (11.7) | 2.4 (7.5) | 0 (2) |
| 74 | Uttaradit | 6979 | 25.4 (2.1) | 129.5 (121.6) | 73.2 (12.5) | 17.8 (13.3) | 10.6 (11.6) | 3.6 (7.6) | 13.7 (12.1) | 7.8 (11.3) | 2.7 (7) | 0.5 (2.2) |
| 75 | Yala | 9710 | 24.9 (1.1) | 160.5 (97.3) | 82.4 (6.2) | 15.2 (19.7) | 9.3 (14.5) | 3.1 (8.7) | 11.4 (16.8) | 6.6 (13.7) | 2 (7.4) | -0.6 (1.9) |
| 76 | Yasothon | 6806 | 26.3 (2.2) | 118.4 (119) | 69.9 (10.1) | 16.9 (12.3) | 10 (9.7) | 3.3 (5.9) | 11.9 (11.5) | 6.7 (9.3) | 1.8 (5.6) | -0.3 (2.3) |

Note: SD: standard deviation; hw.2.95: heatwave with 95^th^ percentile for 2 consecutive days; hw.2.97: heatwave with 97^th^ percentile for 2 consecutive days; hw.2.99: heatwave with 99^th^ percentile for 2 consecutive days; hw.4.95: heatwave with 95^th^ percentile for 4 consecutive days; hw.4.97: heatwave with 97^th^ percentile for 4 consecutive days; hw.4.99: heatwave with 99^th^ percentile for 4 consecutive days; scPDSI: self-calibrated Palmer Drought Severity Index.

8. Vietnam

| **N** | **Province** | **Total dengue** | **Mean monthly temperature (SD)** | **Mean monthly total precipitation (SD)** | **Mean monthly relative humidity (SD)** | **Mean annual hw.2.95 (SD)** | **Mean annual hw.2.97 (SD)** | **Mean annual hw.2.99 (SD)** | **Mean annual hw.4.95 (SD)** | **Mean annual hw.4.97 (SD)** | **Mean annual hw.4.99 (SD)** | **Monthly mean scpdsi (SD)** |
| --- | --- | --- | --- | --- | --- | --- | --- | --- | --- | --- | --- | --- |
| 1 | An Giang | 41250 | 27.1 (1.1) | 157.6 (106.4) | 78.1 (7.8) | 15 (16.4) | 8.4 (11.7) | 3.1 (7.7) | 12 (15.6) | 6.5 (11.8) | 2.7 (7.8) | 0.4 (0.3) |
| 2 | Ba Ria-Vung Tau | 43846 | 26.6 (1) | 172.1 (130.3) | 81.2 (7.1) | 15.3 (13.5) | 8.7 (10.7) | 2.3 (4.9) | 13.2 (12.9) | 6.5 (10.1) | 1.2 (2.6) | -0.8 (0.4) |
| 3 | Bac Giang | 1584 | 22.7 (4.8) | 160.4 (126.4) | 80.3 (6) | 15.7 (11.2) | 9.7 (6.8) | 2.8 (3) | 9.4 (9.5) | 5.1 (4.5) | 0.7 (1.6) | 1.1 (2.1) |
| 4 | Bac Kan | 110 | 20.6 (4.7) | 178.4 (122.7) | 84.1 (4.9) | 15.5 (7.5) | 8.2 (5.7) | 2.9 (2.6) | 6.5 (5.4) | 3.8 (3.5) | 1.2 (2) | 0.5 (2.4) |
| 5 | Bac Lieu | 8342 | 26.9 (1) | 188.3 (125.7) | 80.7 (5.6) | 13.8 (16.1) | 7.9 (11.4) | 2.6 (6) | 10.9 (15.1) | 6.6 (11.5) | 2.3 (6.1) | -0.4 (0.3) |
| 6 | Bac Ninh | 2423 | 23.7 (4.8) | 162.5 (131.4) | 78.1 (5.6) | 15.7 (10.6) | 9.8 (6.8) | 2.7 (2.9) | 9.2 (9.1) | 4.8 (4.6) | 0.7 (1.6) | 1.1 (1.9) |
| 7 | Ben Tre | 20610 | 26.9 (1) | 164.5 (118.7) | 82 (5.5) | 14.7 (13.2) | 8.7 (10.5) | 2 (4) | 11.5 (12.9) | 7.5 (9.9) | 0.9 (3) | -0.5 (0.4) |
| 8 | Binh Dinh | 34512 | 24.9 (2.6) | 166.2 (134.5) | 80.2 (6) | 14.5 (13.8) | 8 (9) | 2.3 (3.7) | 9.5 (11.2) | 5.6 (8.1) | 1.3 (2.9) | -0.6 (1.4) |
| 9 | Binh Duong | 87055 | 27 (1.1) | 166.1 (124.5) | 77.2 (10.8) | 13.6 (11) | 8 (10.1) | 2.4 (5.5) | 10.4 (10.3) | 6.6 (10) | 1.6 (3.9) | -0.7 (0.6) |
| 10 | Binh Phuoc | 33581 | 26.2 (1.2) | 190.1 (147.2) | 76.9 (13.2) | 13.6 (14.8) | 6.9 (12.3) | 2 (4.6) | 10.1 (12.8) | 5.4 (9.9) | 1.3 (3) | -0.9 (0.6) |
| 11 | Binh Thuan | 25669 | 25.5 (1) | 140.1 (97.9) | 79.2 (7.3) | 14.5 (13.7) | 8.5 (11) | 2.4 (4.1) | 10.9 (12.5) | 5.8 (9.9) | 1.6 (3.2) | -0.7 (0.4) |
| 12 | Ca Mau | 22001 | 27 (1) | 203.5 (130.4) | 81.2 (5.7) | 13.5 (15) | 8.4 (11.7) | 2.8 (7.1) | 11.4 (14.4) | 6.9 (11.4) | 1.8 (6) | -0.6 (0.3) |
| 13 | Can Tho | 13660 | 26.9 (1) | 164.7 (111.3) | 80.1 (7) | 14.8 (16.1) | 7.8 (11.6) | 2.9 (7.7) | 12.1 (15.7) | 6.4 (11.3) | 2.4 (7.8) | 0.2 (0.2) |
| 14 | Cao Bang | 59 | 19.7 (4.9) | 152.1 (97.1) | 83.2 (4.7) | 15.3 (8.9) | 8.5 (6) | 2.7 (2.7) | 7.5 (5.6) | 4.5 (4.2) | 1.1 (1.9) | 0.3 (2.6) |
| 15 | Da Nang | 41205 | 24.6 (2.9) | 265.5 (201.7) | 84.7 (4) | 16.6 (17.2) | 10 (13.9) | 3.4 (6) | 11.8 (13.9) | 8.3 (12.9) | 1.9 (5.1) | -0.5 (2.1) |
| 16 | Dak Lak | 52218 | 24.1 (1.7) | 130.1 (91) | 76.5 (7.9) | 14.8 (16) | 8.6 (12.6) | 2.6 (4.5) | 11.3 (14.6) | 4.8 (10.7) | 1.6 (3.7) | -1 (0.8) |
| 17 | Dak Nong | 11666 | 23.2 (1.3) | 157.5 (113.7) | 78.8 (10.5) | 13.7 (15.4) | 6.9 (10.8) | 2.1 (4.3) | 10.7 (13.9) | 4.8 (8.6) | 1 (2.2) | -0.9 (0.6) |
| 18 | Dien Bien | 204 | 20.4 (3.5) | 179.2 (143.2) | 81.7 (7.7) | 15.4 (12.6) | 8.5 (7.5) | 2.6 (5) | 10 (8.9) | 5.3 (6.1) | 1.4 (3.2) | -0.2 (2.7) |
| 19 | Dong Nai | 79315 | 26.3 (1.1) | 187.2 (140.1) | 80.3 (9.5) | 14.8 (12.2) | 8.1 (10.1) | 2.8 (5.3) | 10.6 (11.4) | 6.4 (9.7) | 1.3 (2.2) | -0.7 (0.5) |
| 20 | Dong Thap | 32602 | 27.2 (1) | 155.5 (107.1) | 77.8 (7.8) | 14.7 (15.9) | 8 (11.4) | 2.9 (7.7) | 12 (15.4) | 6.4 (11.3) | 2.4 (7.8) | 0.3 (0.3) |
| 21 | Gia Lai | 41269 | 24 (1.7) | 140.8 (108.7) | 76.9 (8.1) | 14.4 (15.2) | 8.2 (11.2) | 2.7 (4.6) | 10.7 (12.4) | 5.3 (9.8) | 2.1 (4.7) | -0.7 (1.5) |
| **N** | **Province** | **Total dengue** | **Mean monthly temperature (SD)** | **Mean monthly total precipitation (SD)** | **Mean monthly relative humidity (SD)** | **Mean annual hw.2.95 (SD)** | **Mean annual hw.2.97 (SD)** | **Mean annual hw.2.99 (SD)** | **Mean annual hw.4.95 (SD)** | **Mean annual hw.4.97 (SD)** | **Mean annual hw.4.99 (SD)** | **Monthly mean scpdsi (SD)** |
| 22 | Ha Giang | 72 | 19.8 (4.6) | 186.7 (118) | 84.1 (3.8) | 15.3 (8) | 8.2 (5.8) | 2.8 (2.5) | 8.1 (6.1) | 4.7 (4) | 1.1 (1.9) | -0.2 (2.9) |
| 23 | Ha Nam | 1584 | 23.7 (4.7) | 157 (130.9) | 79.8 (5.1) | 15.9 (11.7) | 9.1 (7.3) | 2.7 (3.1) | 11.6 (11) | 5.5 (5.9) | 0.8 (1.8) | 1.2 (1.7) |
| 24 | Ha Tinh | 1319 | 23.5 (4.3) | 191.2 (195.2) | 81.2 (5.9) | 15.5 (16.7) | 9.3 (10.5) | 3.1 (4.1) | 12.4 (15.2) | 4.5 (6.3) | 1.6 (3) | -0.3 (2) |
| 25 | Hai Duong | 1319 | 23.7 (4.8) | 157.5 (129.7) | 79.1 (5.5) | 16.8 (12.6) | 10.1 (8) | 3 (3.1) | 11.2 (10.6) | 7.1 (6.1) | 0.7 (1.6) | 1.4 (1.9) |
| 26 | Hai Phong | 2848 | 23.9 (4.7) | 155.1 (137) | 80 (5.2) | 17.1 (11.9) | 9.6 (8.4) | 2.9 (3) | 12.5 (11.1) | 7.4 (6.6) | 0.8 (1.8) | 1.5 (1.9) |
| 27 | Hanoi | 102536 | 23.6 (4.8) | 158.7 (123.6) | 79 (5) | 15.5 (8.5) | 9.3 (6.8) | 2.6 (2.7) | 8.2 (6.9) | 4.5 (5.3) | 0.8 (1.8) | 0.9 (1.7) |
| 28 | Hau Giang | 4066 | 26.7 (1) | 177.9 (120.3) | 80.9 (6.9) | 13.8 (16.1) | 7.9 (11.4) | 2.6 (6) | 10.9 (15.1) | 6.6 (11.5) | 2.3 (6.1) | -0.1 (0.3) |
| 29 | Ho Chi Minh City | 257305 | 27.1 (1) | 166.3 (120.6) | 78.6 (8.1) | 14.6 (13) | 8.2 (10.2) | 2.2 (4.8) | 11.2 (12.1) | 6.8 (9.9) | 1.6 (4.3) | -0.7 (0.4) |
| 30 | Hoa Binh | 937 | 22.4 (4.6) | 171.7 (140.2) | 83.1 (4.1) | 15.5 (10.3) | 8.3 (6.5) | 2.7 (3.3) | 9.7 (8.2) | 5.5 (5.3) | 0.8 (1.8) | 0.6 (1.4) |
| 31 | Hung Yen | 1597 | 23.8 (4.8) | 155.5 (126.2) | 78.6 (5.4) | 17 (12.2) | 9.6 (8) | 3 (3.2) | 11.3 (10.8) | 6.3 (5.1) | 1.2 (2) | 1.4 (1.8) |
| 32 | Khanh Hoa | 63825 | 24.3 (2) | 172.2 (119.7) | 81.6 (4.6) | 15 (18.1) | 8.2 (10.2) | 2.5 (4.6) | 9.7 (14.3) | 4.5 (8.3) | 1.5 (3.4) | -1.2 (0.5) |
| 33 | Kien Giang | 16166 | 27 (1) | 186.8 (122.9) | 79.9 (6.8) | 14.8 (16.7) | 8.8 (11.6) | 3.1 (8.4) | 12.8 (16.4) | 7.1 (12) | 2.6 (7.5) | 0.2 (0.3) |
| 34 | Kon Tum | 9940 | 21.7 (1.6) | 182.1 (136.8) | 80.6 (9) | 14.5 (15) | 8.5 (10.9) | 2.9 (5.6) | 10.5 (14.1) | 6.3 (9.7) | 2.1 (4.9) | -0.4 (1.9) |
| 35 | Lai Chau | 27 | 19.7 (3.7) | 190.9 (144.5) | 82.2 (6.6) | 15.5 (9.4) | 9.2 (7.6) | 2.4 (4) | 9.2 (8) | 4.8 (7) | 1.6 (3) | -0.5 (2.7) |
| 36 | Lam Dong | 6638 | 21.9 (1) | 173 (110.9) | 81.5 (9.1) | 14.5 (14.3) | 7.8 (11) | 2.2 (4.4) | 10.4 (13.3) | 5.5 (10.7) | 1.1 (2.6) | -0.7 (0.4) |
| 37 | Lang Son | 292 | 21.1 (5) | 154.6 (109.7) | 82.2 (5.8) | 15.5 (11.1) | 9.7 (6.8) | 2.8 (2.9) | 9.2 (9) | 4.8 (4) | 0.7 (1.6) | 0.6 (2.3) |
| 38 | Lao Cai | 243 | 19.7 (4.4) | 178.2 (115.1) | 83.2 (3.7) | 15.4 (9.3) | 8.6 (6.9) | 2.8 (2.6) | 7.9 (6.8) | 4.8 (4.6) | 1.2 (2) | -0.2 (2.8) |
| 39 | Long An | 33065 | 27.2 (1) | 159.6 (113.3) | 77.5 (8.3) | 14.7 (14.2) | 8.1 (10.9) | 2.7 (6) | 11.5 (12.5) | 6.4 (10.5) | 1.9 (5.1) | -0.1 (0.4) |
| 40 | Nam Dinh | 7070 | 24.1 (4.7) | 158.3 (138.1) | 79.7 (5.1) | 16.9 (12) | 9.5 (8.2) | 2.9 (3) | 12.4 (10.8) | 7.3 (6.5) | 1.1 (1.9) | 1.3 (2.1) |
| 41 | Nghe An | 2927 | 22.3 (4.2) | 151.1 (125.7) | 82.2 (4.7) | 15.5 (15.9) | 8.9 (10.3) | 2.6 (4.2) | 11.6 (13.5) | 5.1 (7.2) | 1.4 (3.2) | 0.5 (1.9) |
| 42 | Ninh Binh | 1234 | 23.8 (4.7) | 156.5 (137) | 80.6 (5) | 15.3 (11) | 8.9 (7) | 2.9 (3) | 11.2 (10.2) | 5.5 (5.3) | 0.8 (1.8) | 1.2 (1.8) |
| 43 | Ninh Thuan | 5632 | 24.6 (1.7) | 149.2 (116.8) | 79 (5.6) | 15.2 (17.8) | 8 (10.4) | 2.4 (3.3) | 9.9 (12.3) | 5.5 (8) | 0.9 (2.1) | -0.8 (0.5) |
| **N** | **Province** | **Total dengue** | **Mean monthly temperature (SD)** | **Mean monthly total precipitation (SD)** | **Mean monthly relative humidity (SD)** | **Mean annual hw.2.95 (SD)** | **Mean annual hw.2.97 (SD)** | **Mean annual hw.2.99 (SD)** | **Mean annual hw.4.95 (SD)** | **Mean annual hw.4.97 (SD)** | **Mean annual hw.4.99 (SD)** | **Monthly mean scpdsi (SD)** |
| 44 | Phu Tho | 2231 | 22.9 (4.7) | 174.7 (133.8) | 82.2 (3.7) | 15.6 (8.9) | 9.4 (6.9) | 2.8 (2.8) | 8.5 (6.5) | 4.6 (5.4) | 0.8 (1.8) | 0.2 (1.8) |
| 45 | Phu Yen | 27150 | 25.1 (2.4) | 150.2 (116.6) | 79.8 (5.8) | 15.5 (15.5) | 8.5 (9.8) | 2.2 (3.5) | 11.5 (13.6) | 5.1 (7.6) | 1.2 (2.6) | -1.3 (0.9) |
| 46 | Quang Binh | 20785 | 23.4 (3.8) | 182.2 (186.3) | 82.7 (6) | 16.5 (15.5) | 9.2 (9.7) | 3.5 (4.6) | 12.1 (13.1) | 4.5 (6) | 2.4 (2.9) | -0.9 (2.1) |
| 47 | Quang Nam | 31823 | 23.3 (2.8) | 231.3 (170.5) | 83 (5.5) | 15 (14.7) | 7.9 (8.8) | 2.4 (3.8) | 8.9 (10.2) | 5.5 (7.3) | 1.3 (2.9) | -0.6 (2.1) |
| 48 | Quang Ngai | 15997 | 24.4 (2.8) | 238 (194.9) | 82 (5.8) | 15 (16) | 8.2 (9.6) | 2.2 (3.5) | 11 (12.4) | 5.7 (8.1) | 1.3 (2.9) | -0.4 (1.9) |
| 49 | Quang Ninh | 2452 | 22.4 (4.7) | 173.2 (145.7) | 82.2 (6) | 16.6 (12.5) | 10.1 (7.9) | 3.2 (3) | 10.9 (10.6) | 7 (6.4) | 0.8 (1.8) | 1 (2.1) |
| 50 | Quang Tri | 10175 | 24 (3.2) | 200.5 (178.4) | 82.5 (6.6) | 16.2 (16.4) | 10 (12.2) | 3.5 (5) | 12.1 (14.2) | 7.7 (9.7) | 2.4 (3.4) | -1.6 (2.1) |
| 51 | Soc Trang | 18427 | 26.8 (1) | 173.7 (120.6) | 81.2 (5.5) | 14.3 (15.9) | 7.8 (11.2) | 2.9 (6.3) | 12 (14.3) | 6.8 (11.2) | 2.1 (5.5) | -0.3 (0.4) |
| 52 | Son La | 325 | 20.4 (3.9) | 145 (118.9) | 80.7 (5.7) | 15.3 (12) | 9.2 (8.5) | 2.7 (4.2) | 9.1 (7.7) | 6.1 (6.2) | 0.8 (2.7) | 0.4 (2.4) |
| 53 | Tay Ninh | 22346 | 27.5 (1.2) | 154.5 (117.6) | 74.7 (11.2) | 14.2 (11.6) | 8.3 (9.8) | 2.7 (5.6) | 10.8 (11.2) | 6.5 (9.7) | 1.6 (3.9) | 0 (0.6) |
| 54 | Thai Binh | 1697 | 24.1 (4.7) | 159.6 (139.1) | 79.5 (5.2) | 17.1 (12.3) | 9.6 (8.4) | 2.9 (3) | 12.5 (11.5) | 7.4 (6.5) | 1.1 (1.9) | 1.4 (2) |
| 55 | Thai Nguyen | 759 | 22.4 (4.7) | 175.3 (130.7) | 81.7 (5.6) | 15.2 (8.2) | 9.5 (6.8) | 2.5 (2.5) | 7.9 (6.3) | 4.5 (5.3) | 0.8 (1.8) | 0.7 (2) |
| 56 | Thanh Hoa | 5734 | 22.7 (4.5) | 155.6 (130.2) | 82.5 (4.2) | 15.5 (12.9) | 8.6 (8) | 2.5 (3.9) | 12.4 (10.9) | 5.7 (6.1) | 0.7 (1.6) | 0.8 (1.4) |
| 57 | Thua Thien - Hue | 8403 | 24 (3) | 260.5 (200) | 84.2 (5.4) | 16.6 (17.7) | 10.2 (13.8) | 3.6 (6.5) | 11.7 (15.8) | 6.9 (11.2) | 1.4 (3.2) | -0.9 (2.3) |
| 58 | Tien Giang | 31222 | 26.9 (1) | 164.3 (117.3) | 80.5 (6.8) | 14.7 (13.2) | 8.5 (10.4) | 2.5 (5.6) | 10.9 (12.6) | 7.6 (10.3) | 1.8 (4.9) | -0.4 (0.4) |
| 59 | Tra Vinh | 11390 | 26.8 (1) | 165.5 (118) | 81.7 (5.1) | 15.2 (14.6) | 8.7 (10.7) | 2.7 (6.1) | 12.2 (13.7) | 7 (10.4) | 2.4 (5.6) | -0.5 (0.5) |
| 60 | Tuyen Quang | 542 | 22.1 (4.6) | 193.1 (142.3) | 83.3 (4.3) | 15.7 (7.3) | 8.2 (5.3) | 2.8 (2.7) | 7.4 (5.8) | 3.8 (4.3) | 1.5 (2.2) | 0 (2.5) |
| 61 | Vinh Long | 13032 | 26.8 (1) | 166.8 (116.6) | 81.6 (6.7) | 14.8 (14.9) | 8.6 (11.3) | 2.9 (7.2) | 12.5 (13.7) | 6.5 (11) | 2.5 (7.2) | -0.2 (0.3) |
| 62 | Vinh Phuc | 1243 | 23.4 (4.7) | 159.7 (126.2) | 80.4 (4.6) | 15.5 (8.3) | 9.2 (6.4) | 2.7 (2.7) | 7.8 (6.2) | 4.5 (5.2) | 0.8 (1.8) | 0.7 (1.8) |
| 63 | Yen Bai | 486 | 20.6 (4.4) | 224.4 (154.4) | 83.5 (3.3) | 15.4 (10.6) | 8.8 (7.4) | 2.5 (2.5) | 9.7 (7.8) | 5.3 (5.6) | 0.7 (1.6) | - 1. (2.5) |

Note: SD: standard deviation; hw.2.95: heatwave with 95^th^ percentile for 2 consecutive days; hw.2.97: heatwave with 97^th^ percentile for 2 consecutive days; hw.2.99: heatwave with 99^th^ percentile for 2 consecutive days; hw.4.95: heatwave with 95^th^ percentile for 4 consecutive days; hw.4.97: heatwave with 97^th^ percentile for 4 consecutive days; hw.4.99: heatwave with 99^th^ percentile for 4 consecutive days; scPDSI: self-calibrated Palmer Drought Severity Index.
