# Supplementary material for: Extreme weather events and dengue in Southeast Asia: A regionally-representative analysis of 291 locations from 1998 to 2021
Source: PLoS Negl Trop Dis. 2025 Sep 4;19(9):e0012649. doi: 10.1371/journal.pntd.0012649 (PMC12419652; doi:10.1371/journal.pntd.0012649)
Supplement: S4 Table — (DOCX) [file pntd.0012649.s005.docx]

**S4 Table. Generalized cross-validation score of base dengue model with varying seasonality functions.**

We first fitted the base dengue model using various seasonality control functions including smoothing spline with 3df, 4df, 5df, cyclic cubic regression spline, indicator of month, and one pair of sine and cosine function. A quasi-Poisson distribution was assumed to account for potential over-dispersion in the dengue data. The logarithm of the total population for each location was included as an offset to adjust dengue case count by population. Long-term trends and interannual variability were accounted for incorporating an indicator for each year in the time series. The seasonality control function was selected based on the lowest Generalized Cross-Validation (GCV) score in the base dengue model. Dengue base model can be represented with the equation:

$$\mathrm{Log} {(Y}_{i,t})=\alpha+\mathrm{Log} \left( P_{i,t} \right)+s\left( t \right)+\delta_{i,t}+ \nu_{i}$$

Where $Yt$ : represents monthly dengue case following a quasi-Poisson distribution with overdispersion;

$\alpha$ : indicates the intercept.

$\log\left( P_{i,t} \right)$ : represents logarithm of population per location in each month as an offset.

$s\left( t;\beta\right)$ : denotes the smooth seasonal trend defined by smoothing splines of time with 5 df.

$\delta_{i,t}$ : designates long-term trend using indicator variables for each year in each location.

$\nu_{i}$ : expresses unstructured random effects for each location.

| **Seasonality function** | **GCV** |
| --- | --- |
| Smoothing spline with 3 df | 106.34 |
| Smoothing spline with 4 df | 104.91 |
| Smoothing spline with 5 df | 104.68 |
| Cyclic cubic regression spline | 116.01 |
| 1 pair of sine and cosine function | 118.73 |

Note: Red color represents model selected; GCV, generalized cross validation;

df, degree of freedom
