# Supplementary material for: Extreme weather events and dengue in Southeast Asia: A regionally-representative analysis of 291 locations from 1998 to 2021
Source: PLoS Negl Trop Dis. 2025 Sep 4;19(9):e0012649. doi: 10.1371/journal.pntd.0012649 (PMC12419652; doi:10.1371/journal.pntd.0012649)
Supplement: S5 Table — (DOCX) [file pntd.0012649.s006.docx]

# **S5 Table. Generalized cross-validation score of varying cross basis specification in main model.**

Note: Red color represents model selected; GCV, generalized cross validation; NS, natural cubic spline; HW, monthly number of heatwave days; scPDSI, self-calibrated Palmer drought severity index; df, degree of freedom

| **Specification of cross basis function** | **HW-dengue** | **scPDSI-dengue** |
| --- | --- | --- |
| NS with 3 df for exposure and 3 df for lag | NA | 92.81 |
| NS with 2 Equally space internal knot for exposure and 3 df for lag | 89.98 | 92.83 |
| NS with 2 internal knot at 25th and 75th for exposure and 3df lag | 90.62 | 92.82 |
| NS with 1 internal knot at 50th for exposure and 3df lag | 90.62 | 92.85 |
