# Supplementary material for: Extreme weather events and dengue in Southeast Asia: A regionally-representative analysis of 291 locations from 1998 to 2021
Source: PLoS Negl Trop Dis. 2025 Sep 4;19(9):e0012649. doi: 10.1371/journal.pntd.0012649 (PMC12419652; doi:10.1371/journal.pntd.0012649)
Supplement: S6 Table — (DOCX) [file pntd.0012649.s007.docx]

# **S6 Table. Pooled relative risks of heatwave-dengue association, relative to month with no heatwaves.**

| **Monthly number of heatwaves** | **RR (95% CI)** |
| --- | --- |
| 5 days | 1.25 (1.18–1.32) |
| 7 days | 1.28 (1.19–1.38) |
| 15 days | 0.74 (0.66–0.82) |
| 21 days | 0.49 (0.42–0.57) |
| 30 days | 1.34 (0.89–2.01) |

Note: RR: Relative Risk; CI: Confidence Interval; SEA, Southeast Asia
