# Supplementary material for: Extreme weather events and dengue in Southeast Asia: A regionally-representative analysis of 291 locations from 1998 to 2021
Source: PLoS Negl Trop Dis. 2025 Sep 4;19(9):e0012649. doi: 10.1371/journal.pntd.0012649 (PMC12419652; doi:10.1371/journal.pntd.0012649)
Supplement: S7 Table — (DOCX) [file pntd.0012649.s008.docx]

# **S7 Table. The relative risks at each lag up to 4 months of heatwave-dengue association, relative to month with no heatwaves.**

Note: HW, heatwave; RR, relative risk; CI, confidence interval, SEA, Southeast Asia.

| **Lag** | **5 HW days/month**  **RR (95% CI)** | **15 HW days/month**  **RR (95% CI)** | **30 HW days/month**  **RR (95% CI)** |
| --- | --- | --- | --- |
| Lag 0 | 0.85 (0.83–0.88) | 0.67 (0.63–0.71) | 0.48 (0.38–0.61) |
| Lag 1 | 0.96 (0.94–0.97) | 0.76 (0.74–0.79) | 0.70 (0.62–0.78) |
| Lag 2 | 1.06 (1.04–1.08) | 0.90 (0.87–0.92) | 1.03 (0.92–1.15) |
| Lag 3 | 1.15 (1.13–1.16) | 1.11 (1.08–1.14) | 1.57 (1.44–1.70) |
| Lag 4 | 1.23 (1.21–1.26) | 1.42 (1.38–1.47) | 2.43 (2.20–2.68) |
