# Supplementary material for: Extreme weather events and dengue in Southeast Asia: A regionally-representative analysis of 291 locations from 1998 to 2021
Source: PLoS Negl Trop Dis. 2025 Sep 4;19(9):e0012649. doi: 10.1371/journal.pntd.0012649 (PMC12419652; doi:10.1371/journal.pntd.0012649)
Supplement: S8 Table — (DOCX) [file pntd.0012649.s009.docx]

# **S8 Table. Pooled relative risks of scPDSI-dengue association, relative to a scPDSI value of zero.**

| **scPDSI** | **Value** | **RR (95% CI)** |
| --- | --- | --- |
| Drought condition | –4 | 1.85 (1.73–1.99) |
| Extremely wet condition | 4 | 0.92 (0.87–0.96) |
| Severely wet condition | 8.1 | 1.20 (0.96–1.51) |

Note: scPDSI, self-calibrated Palmer Drought Severity Index; RR, relative risk; CI, confidence interval; SEA, Southeast Asia
