# Supplementary material for: Extreme weather events and dengue in Southeast Asia: A regionally-representative analysis of 291 locations from 1998 to 2021
Source: PLoS Negl Trop Dis. 2025 Sep 4;19(9):e0012649. doi: 10.1371/journal.pntd.0012649 (PMC12419652; doi:10.1371/journal.pntd.0012649)
Supplement: S9 Table — (DOCX) [file pntd.0012649.s010.docx]

# **S9 Table. The relative risks at each lag up to 4 months of scPDSI-dengue association, relative to a scPDSI value of zero.**

| **Lag** | **Drought condition (**–**4)**  **RR (95% CI)** | **Extremely wet condition (4)**  **RR (95% CI)** |
| --- | --- | --- |
| Lag 0 | 1.13 (1.05–1.22) | 0.96 (0.90–1.03) |
| Lag 1 | 0.95 (0.92–0.98) | 0.95 (0.92–0.97) |
| Lag 2 | 0.92 (0.86–0.98) | 0.95 (0.90–1.01) |
| Lag 3 | 1.13 (1.10–1.18) | 0.99 (0.98–1.02) |
| Lag 4 | 1.62 (1.49–1.76) | 1.05 (0.98–1.12) |

Note: RR, relative risk; CI, confidence interval, SEA, Southeast Asia.
