# Supplementary material for: Extreme weather events and dengue in Southeast Asia: A regionally-representative analysis of 291 locations from 1998 to 2021
Source: PLoS Negl Trop Dis. 2025 Sep 4;19(9):e0012649. doi: 10.1371/journal.pntd.0012649 (PMC12419652; doi:10.1371/journal.pntd.0012649)
Supplement: S1 Fig — (DOCX) [file pntd.0012649.s011.docx]

**S1 Fig** Flowchart summarizing the data collection and modeling steps.

Climate and dengue data collection

Data harmonization

Fitting base dengue model

Fitting main model

Sensitivity analysis

Data cleaning

Descriptive analyses

Data preparation

Statistical analysis
