# Supplementary material for: Extreme weather events and dengue in Southeast Asia: A regionally-representative analysis of 291 locations from 1998 to 2021
Source: PLoS Negl Trop Dis. 2025 Sep 4;19(9):e0012649. doi: 10.1371/journal.pntd.0012649 (PMC12419652; doi:10.1371/journal.pntd.0012649)
Supplement: S3 Fig — (DOCX) [file pntd.0012649.s013.docx]

**S3 Fig. Time-series plot of total yearly dengue cases in eight Southeast Asia countries.**
