# Supplementary material for: Extreme weather events and dengue in Southeast Asia: A regionally-representative analysis of 291 locations from 1998 to 2021
Source: PLoS Negl Trop Dis. 2025 Sep 4;19(9):e0012649. doi: 10.1371/journal.pntd.0012649 (PMC12419652; doi:10.1371/journal.pntd.0012649)
Supplement: S4 Fig — (DOCX) [file pntd.0012649.s014.docx]

**

# **S4 Fig. Pearson’s correlation coefficients among dengue and climate variables.**

Note: Temp, Monthly mean temperature; Preci, Monthly total precipitation; RH, Monthly mean relative humidity; HW, total of number of heatwave days per month using definition of 95^th^ percentile for 2 consecutive days.
