# Supplementary material for: Extreme weather events and dengue in Southeast Asia: A regionally-representative analysis of 291 locations from 1998 to 2021
Source: PLoS Negl Trop Dis. 2025 Sep 4;19(9):e0012649. doi: 10.1371/journal.pntd.0012649 (PMC12419652; doi:10.1371/journal.pntd.0012649)
Supplement: S5 Fig — (DOCX) [file pntd.0012649.s015.docx]

# **S5 Fig. Time series plot of monthly dengue and climate variables for the selective locations with the highest number of total dengue case for each country.**

Heatwave definition with 95^th^ percentile for 2 consecutive days.
