# Supplementary material for: Extreme weather events and dengue in Southeast Asia: A regionally-representative analysis of 291 locations from 1998 to 2021
Source: PLoS Negl Trop Dis. 2025 Sep 4;19(9):e0012649. doi: 10.1371/journal.pntd.0012649 (PMC12419652; doi:10.1371/journal.pntd.0012649)
Supplement: S9 Fig — (DOCX) [file pntd.0012649.s019.docx]

**S9 Fig. Sensitivity analysis result of using different seasonality functions for heatwave-dengue, and scPDSI-dengue associations.**
