# Supplementary material for: Extreme weather events and dengue in Southeast Asia: A regionally-representative analysis of 291 locations from 1998 to 2021
Source: PLoS Negl Trop Dis. 2025 Sep 4;19(9):e0012649. doi: 10.1371/journal.pntd.0012649 (PMC12419652; doi:10.1371/journal.pntd.0012649)
Supplement: S12 Fig — (DOCX) [file pntd.0012649.s022.docx]

# **S12 Fig. Sensitivity analysis result of conducting analysis with data from 1998–2019 (excluding covid year).**
