# Supplementary material for: Extreme weather events and dengue in Southeast Asia: A regionally-representative analysis of 291 locations from 1998 to 2021
Source: PLoS Negl Trop Dis. 2025 Sep 4;19(9):e0012649. doi: 10.1371/journal.pntd.0012649 (PMC12419652; doi:10.1371/journal.pntd.0012649)
Supplement: S14 Fig — (DOCX) [file pntd.0012649.s024.docx]

# A. Sensitivity analysis result of excluding Cambodia data

# ****

# B. Sensitivity analysis result of excluding Laos data

**S14 Fig. Sensitivity analysis result of excluding Cambodia and Laos data.**
