# Supplementary material for: Extreme weather events and dengue in Southeast Asia: A regionally-representative analysis of 291 locations from 1998 to 2021
Source: PLoS Negl Trop Dis. 2025 Sep 4;19(9):e0012649. doi: 10.1371/journal.pntd.0012649 (PMC12419652; doi:10.1371/journal.pntd.0012649)
Supplement: Attachment — Submitted filename: Response to reviewers.docx [file pntd.0012649.s026.docx]

**Response to reviewers**

Reviewer #1

| Reviewer’s comment | Author’s response |
| --- | --- |
| 1. The figures lack simplicity for a non expert reader. Maybe some clarifications could be made | Thank you very much for your reviews and comments. We have addressed your comments below:  We have clarified the explanations and captions to make the meaning of the figures more immediately apparent to non-experts. |
| 2. I think the summary conclusion "Our finding offer stakeholders sizeable amount of time to organize and implement public health interventions in minimizing the prospective dengue risks" need to be rewritten in a more rigorous way underscoring the quantitative study outcomes. | We have revised this in the manuscript line 54-55.  *“…The findings highlight the need for public health interventions to mitigate the potential dengue risks posed by EWEs in the context of climate change in SEA…”* |
| 3. Confirm the contributions of the impressive co-authors list ie from 18 institutions vs acknowledgments. | We confirm that all authors made substantial contributions to the study, including the collection and provision of dengue and climate data, engaging in discussions, offering valuable feedback on the results section, and reviewing the final manuscript. |

Reviewer #2

| Reviewer’s comment | Author’s response |
| --- | --- |
| This study was very well presented. The study’s idea was also clear and was practical for epidemiological and community health purpose. I can see that the authors did many attempts in statistical analyses to check every possibilities those might affect the results of the study. The results were also explained well in the discussion section.  To improve this article, there are some issues authors need to elaborate. Those are: 1. Table 1: data for each country covered different time period, e.g. Cambodia 1998-2010 while Indonesia 2010-2020. As we all know, ideally data must be collected in similar time period. Different timing in data collection may potentially lead to ’temporal bias’. Is there any analysis/strategy authors provided to overcome this period differences? | Thank you very much for your reviews and comments. We have addressed your comments below:  Our primary assumption is that the underlying risk function remains broadly similar across different times and locations, provided that the model adequately accounts for these variabilities. Period differences are addressed through the inclusion month and year terms to capture long-term temporal variations.  However, Cambodia is the only country without an overlapping data collection period with others. To address this, we conducted a sensitivity analysis excluding Cambodia data to assess its potential impact on the final results (Supplementary Figure S14). The analysis demonstrated no significant differences compared to the models that included Cambodia data, indicating minimal influence on the overall outcomes. |
| 2. Different mosquito species have different characteristic. From a literature (<http://www.parasitesandvectors.com/content/7/1/338>), we could learn that temperature does not limit the persistence of Ae. albopictus. In this study, authors analysed the dengue incidence in association with selected EWEs. Providing data about the dominant mosquito species in each region will be a valuable addition to the explanation/discussion. | We have added this information in the manuscript line 93–94:  *“…Given that Ae. aegypti is the main vector of dengue fever in Southeast Asia, rising temperatures pose a real threat of increased dengue risk [13,14]…”* |
| 3. Is it possible that each country gave different result when analysed separately? As each country might have different weather variability and different vector control strategy (e.g. some countries apply ‘closing, draining, and burying water-filled container’. This behaviour might reduce dengue incidence even in drought/ heatwave). | It is possible that analyzing each country separately could yield different results. However, the primary objective of this study was to assess the overall effect of extreme weather events (EWEs) on dengue in the Southeast Asia (SEA) region, rather than to compare results across individual countries.  The chosen modeling approach provided the best overall fit for the region but may not have been optimal for each specific location or country. We also noted this in the limitations line 315–318:  *“….First, a significant limitation is the use of a single set of modeling parameters across all the locations, which may heavily impact location/country-specific estimations. Specifically, the chosen modeling may not fit the data well in certain locations, even if it results in the best overall fit.”* |

Reviewer #3

| Reviewer’s comment | Author’s response |
| --- | --- |
| 1. The limitations related to the ecologic design should be acknowledged. | Thank you very much for your reviews and comments. We have addressed your comments below:  We have added the limitation related to ecologic design in manuscript line 328–330:  *“…Due to the ecological design of our study, there is a potential for ecological fallacy, which the findings at the regional level may not accurately reflect the outcomes for individual countries or specific locations.…”* |
| 2. Extreme weather events have become more frequent. They have occurred on a worldwide scale. Analysis of its association with the occurrence of vector-borne diseases should be encouraged. However, local factors may have significant importance in this relationship. | Local factors may have significant importance in this relationship. However, we did not include any explicit local factors in the model because the main objective of our study is to investigate the regional patterns rather investigate the effect of specific local factors on the climate-dengue association. In addition, we accounted for the variability of local factors on the relationship by including a random effect term in our model. This term captures variability across locations and addresses unobserved heterogeneity among them. Further research is needed to explore the role of specific local factors in shaping this association is a different set of analyses (i.e. effect modification). We have added this in manuscript line 330–334:  *“…Although factors such as urbanization and dengue control measures may influence or modify the impact of EWEs on dengue risk, we did not include these variables in our models as the study focused on the overall relationship between EWEs and dengue. Future research should explore how these factors modify the relationship between EWEs and dengue risk to provide a more comprehensive understanding of their interactions….”* |

Reviewer #4

| Reviewer’s comment | Author’s response |
| --- | --- |
| 1. The dengue data collection periods vary across countries, with some data not covering the full span from 1998 to 2021. For instance, only two provinces in Lao PDR are included. This raises questions about potential biases or limitations in the results. The manuscript should clarify the rationale behind these inconsistencies and assess their impact on the study's findings. | Thank you very much for your reviews and comments. We have addressed your comments below:  Our primary assumption is that the underlying risk function remains broadly similar across different times and locations, provided that the model adequately accounts for these variabilities. Period differences are addressed through the inclusion month and year terms to capture long-term temporal variations.  In addition, our aim was to make the results as representative of the Southeast Asia (SEA) region as possible, which is why we included data from as many locations as feasible. Notably, Cambodia was the only country with a non-overlapping data collection period and Laos was the only country with only two provinces. To address this potential limitation, we performed a sensitivity analysis excluding Cambodia and Laos data to evaluate its impact on the final results (Supplementary Figure S14). The analysis revealed no significant differences compared to the model that included Cambodia and Laos, suggesting minimal influence on the overall outcomes. |
| 2. While the methodological details are well-presented, the inclusion of a flowchart summarizing the data collection and modeling steps would enhance transparency and aid reader comprehension. | We have included a flowchart summarizing the data collection and modeling steps in Supplementary Figure S1. |
| 3. Prolonged heatwaves often lead to drought conditions. The manuscript should explore whether there is any overlap or interaction between these two EWEs and how this might influence dengue risk. | Heatwaves are short-term events that typically last for days, whereas droughts are long-term phenomena that can persist for months or even years. Additionally, while heatwaves are solely driven by temperature, droughts are influenced by both temperature and precipitation levels. However, we acknowledge that there could be compound extreme weather events that heatwaves happen during drought conditions. Our analysis did not explore this interaction for several reasons. First, our primary objective was to assess the independent contributions of each EWE to dengue risk. Second, the complex nature of interactions between heatwaves and droughts would require additional modeling approaches and datasets to accurately capture their combined effects, which were beyond the scope of this study. Nevertheless, we recognize the importance of understanding these interactions and suggest that future research could delve deeper into how overlapping periods of heatwaves and droughts influence dengue transmission dynamics. |
| 4. The descriptions of **Figure 2B**, **Figure 3B**, and **Figure 3C** (Page 9, lines 188-190, 202-206; Page 11, lines 245-255) appear unclear or inaccurate. The manuscript would benefit from specifying and clearly labeling the coordinate axes to improve interpretability. | We have made changes to the description accordingly in line 207–208 and in line 221–226:  line 207–208: *“…Figure 2B indicates lag-response association curves of specific monthly number of heatwave days. Month with 7 and 21 heatwave days increase dengue risk after lag 2 and 3 months, respectively.…”*  Line 221–226: *“…Figure 3B indicates association curves of specific scPDSI and dengue across all lag periods. Drought conditions (scPDSI≤–4) shows both immediate and delayed positive association on dengue risk within lag 0 and after two-months lag. Extremely wet conditions (scPDSI=4) show an increased dengue risk at three-months lag, suggesting a delayed positive effect on dengue risk. Figure 3C illustrates the association between scPDSI and dengue risk across all lags. Notably, when the scPDSI exceeds 5·8, there is an increased dengue risk observed at a one month and after a three-month lag.…”* |
| 5. On Page 10-11, lines 230-232, the manuscript explains the decline in dengue risk during months with more than 12 heatwave days due to reduced mosquito survival and feeding activity. However, the subsequent increase in dengue risk during months with over 27 heatwave days is not adequately addressed. The authors should provide a plausible explanation for this observation. | We did not provide a detailed discussion on the observed increase in dengue risk during months with over 27 heatwave days due to limited evidence supporting this finding (confidence interval indicate the result was not significant). The marginal effect may result from the very low number of months with a high frequency of heatwaves, which weakens the strength of the evidence. However, this increase might be attributed to the rise in dengue risk after a two-month lag. Further research with collecting data from more locations, is needed to provide a more robust understanding of this relationship at the extreme number of heatwave. We have added this explaination in the manuscript in line 254–258:  *“…In addition, while our study showed an increase in dengue risk following months with 27 heatwave days, the result is marginal due to the low number of months with an extreme frequency of heatwave days. This increase could potentially be attributed to the delayed effect of heatwaves on dengue risk. Further research is needed to investigate the impact of an extreme number of heatwave days in greater detail.…”* |
| 6. The statement on Page 11, line 253, that "extremely wet conditions show a reduction in dengue risk" is misleading. An increase in dengue risk is observed when the scPDSI exceeds 5.8, even if the result is not statistically significant. This should be rephrased for accuracy. | We have revised accordingly in the manuscript in line 278–280.  *“…scPDSI index and dengue has a non-linear association with drought conditions showing an increase in dengue risk and extremely wet conditions at scPDSI value between 4 and 5.8 showing a reduction in dengue risk….”* |
| 7. On Page 12, lines 257-258, the manuscript notes that mosquito eggs can survive up to 120 days in drought conditions. However, Figure 3B shows a rising relative risk (RR) of dengue after a lag of more than three months, with greater increases over time. The authors should reconcile these findings and provide a detailed explanation. | Mosquito eggs can remain viable for up to 120 days in drought conditions, enabling them to hatch when conditions become favorable, such as after rainfall or when water is available. This biological resilience may lead to an accumulation of viable eggs that hatch simultaneously, increasing the adult mosquito population and subsequently dengue risk after a delayed period. The rising RR observed in Figure 3B beyond the three-month lag could reflect this delayed effect, where prolonged drought is followed by conditions that facilitate mosquito hatching and breeding. For example, human behavioral factors, such as water storage during droughts, could further support mosquito breeding once the drought ends.  We have updated the manuscript to include this explanation in line 283–287.  *“…Mosquito eggs can remain viable for up to 120 days during drought conditions, hatching simultaneously when favorable conditions, such as the return of water availability, are restored [9]. The observed three-month lag between extreme drought events and an increase in dengue risk may stem from gradual changes in domestic water storage practices as households adapt to water scarcity.…”* |

Reviewer #5

| Reviewer’s comment | Author’s response |
| --- | --- |
| On the other hand, the methods seem to have some limitations. The greatest concern is the absence of considering the "multiple" comparisons problem. Classical significance tests fit well to a single, pre-specified test. The methods in this paper did not indicate which, if any, parts of the specification had been chosen before the authors started their analysis, and how their revised their approach based on their interim results. As is well known, the more tests that are performed, the greater the chance that some "significant" result can be found by chance. In this study, apparent opportunities for multiple comparison could arise in many ways, such as:  (1) The choice of the statistical model. The authors chose smoothed splines. They could have chosen averages, a specific form, a model incorporating the month of the year as an independent variable, or some other structure.  (2) The specific parameters within the chosen model. For example, the spline used 5 degrees of freedom. The sensitivity analyses considered a small limited range but not a full defense against multiple comparisons;  (3) The definition of the heat index. The authors give citations 18 and 19, but do not say whether they explicitly followed those prior definitions.  (4) The drought index. While the use of an established index is a virtue, they authors did not give a prior endorsement as to why an index developed for agriculture would apply to dengue risk. | Thank you very much for your reviews and comments. We have addressed your comments below:  (1) We developed our model based on prior knowledge and exploratory analysis. Previous literature has demonstrated that temperature and precipitation exhibit non-linear effects on dengue transmission. A smoothed spline function was selected for its flexibility in capturing these complex non-linear relationships. Also, dengue is strongly influenced by seasonality. To incorporate this, our model explicitly accounts for seasonality by including a seasonal term. We began by fitting the seasonality component of the model, using the Generalized Cross-Validation (GCV) score as the criterion to identify the best-fitting model.  (2) The decision to use 5 degrees of freedom for the spline function to account for seasonality was guided by visual inspection of dengue boxplot data and exploratory analysis. The choice of this modeling specification reflects a balance between achieving a good model fit and maintaining interpretability. Given that our dengue data is on a monthly scale, using too few degrees of freedom would fail to adequately capture the seasonal variations in dengue incidence. Conversely, using too many degrees of freedom could result in overfitting and make the model results more difficult to interpret. We conducted various sensitivity analysis and reported the result in the supplementary documents to ensure the transparency of our modeling choice.  (3) There is no universal definition for heatwave. Typically, it is often described as a period of consecutive days with temperatures surpassing a defined threshold. This threshold can be absolute (grounded in specific temperature values) or relative (based on the temperature within the community). In this research, heatwaves were defined using relative thresholds, which consider each location’s own long-term daily mean temperature. This method enables regional adaptation by comparing temperature to the norm for each specific location. A heatwave was characterized by two or more consecutive days with a daily mean temperature surpassing the 95th percentile of its distribution in each specific location. This definition has also been utilized in previous research examining the effects of heatwaves on health outcomes. To ensure the robustness and transparency of our findings, we conducted multiple sensitivity analyses using alternative heatwave definitions, addressing the lack of a universally accepted definition.  (4) While scPDSI is an agricultural drought index, it reflects broader hydrological and meteorological patterns that influence vector-borne disease dynamics. scPDSI index relies on key meteorological inputs such as precipitation, temperature, and soil moisture capacity. Precipitation data represents the total water input, while temperature data is used to calculate potential evapotranspiration (PET), which measures how much water could evaporate or transpire if water were fully available. Soil moisture capacity, determined by local soil characteristics, indicates how much water the soil can hold. Therefore, it serves as a proxy for environmental conditions that affect mosquito ecology and, consequently, dengue transmission. Previous studies also used scPDSI to quantify the effect of hydrometeorological condition on dengue in Brazil and China. (Lowe R. et al, 2021; Li C. et al, 2023) |
| Additional concerns relate to the exclusion of potentially relevant variables. The authors explicitly acknowledged the exclusion of dengue control measures, which might have been very relevant However, they also excluded time and items associate with it, such as increasing national per capita income, degree of urbanization (as dengue is a primarily urban disease, and variations among sites. The authors' hypothesis around drought responses would seem especially affected by these omitted variables. Higher income and more urban environments might be more likely to use air conditioning or have household members spending time in such settings working, studying, or shopping. Likewise, storing water in unsealed containers would apply most to locations mostly in lower income countries.  If the authors were able to incorporate some of these variables in their models their paper would be strengthened. If they cannot, they may wish to acknowledge that limitation and mention some of the topics as promising areas for further research. | We acknowledge that the result might be affected by the local variable including national per capita income, urbanization, etc… However, since the objective of this study was to investigate the overall effect of EWEs on dengue risk in SEA region and not to investigate the effect of specific local factors on the climate-dengue association, we did not include any explicit local factors in the model. We accounted for the influence of local factors on the relationship by including a random effect term in our model. This term captures variability across locations and addresses unobserved heterogeneity among them. Further research is needed to explore the role of specific local factors in shaping this association. We have added the above limitation in manuscript line 329–333:  *“…Although factors such as urbanization and dengue control measures may influence or modify the impact of EWEs on dengue risk, we did not include these variables in our models as the study focused on the direct relationship between EWEs and dengue. Future research should explore how these factors modify the relationship between EWEs and dengue risk to provide a more comprehensive understanding of their interactions…”* |
| It was not clear why the variability in dengue reporting was a factor. Passive dengue reporting is known to capture only a portion of dengue cases. Generally the proportion increases with the quality of the country's health system (see papers by Shepard DS, Undurraga E and colleagues). But if that fraction were consistent over time, it would be captured in the location-specific constant. | We acknowledge that if dengue reporting has remained consistent over time, it would be captured by the location-specific constant. Furthermore, any improvements in surveillance system coverage over time would be accounted for by the long-term trends incorporated in the model. Therefore, variability in reporting is unlikely to significantly affect the validity of this study.  However, variability in dengue reporting across countries is influenced by differences in case definitions. While some countries rely on laboratory-confirmed cases, others use symptomatic criteria (see Supplementary Document Table S1). These discrepancies may lead to over- or underreporting, affecting the accuracy of dengue case counts and potentially misrepresenting the true burden of the disease. |
| Despite the noted strengths, this reviewer is concerned about the effect of the limitations in the methods on the conclusions. Trying to slow long term global warming may be a long term approach. Based on the study's complex relationships, it would be helpful to summarize the next impact of global warming on dengue cases. | Our study offers a baseline understanding of the risks that could serve as platform to understanding the future risks posed by a changing climate. This was stated in the conclusions of manuscript line 336-338:  *“…The findings highlight the need for public health interventions to mitigate the potential dengue risks posed by EWEs in the context of climate change in SEA.”* |
| I would encourage the authors to clarify their purpose of their study by inserting the word "cases" after "dengue" and the adjective "exploratory) before the words "regionally- representative..." in the title of their manuscript. | We appreciate the reviewer’s suggestion to revise the title for clarity. However, we would like to respectfully clarify our perspective regarding this change. The current title is intentionally broad to reflect the overarching focus of our study, which extends beyond individual "cases" of dengue to broader patterns and implications at a regional level. Adding the word "cases" might narrow the scope and misrepresent the comprehensive nature of our analysis.  Similarly, while we acknowledge the value of emphasizing the exploratory nature of the study, we believe that including the adjective "exploratory" in the title may unnecessarily downplay the robustness of our methodological approach and findings. The study is regionally representative and based on rigorous analysis, which we feel is appropriately conveyed in the current title.  We hope the reviewer understands our rationale for maintaining the title as it stands. However, we remain open to further discussion or alternative suggestions to improve clarity without altering the scope or perceived rigor of the study. |
| line 59. Change "Flaviviruses" to lower case. line 80. After "2 to 10 days" insert the time period (e.g. annually). line 84. After "limited," insert a comma. line 139. Inset "a" before "previous study" line 172. Insert "The" before "State of Selangor line 178. Column for precipitation. Add units to label (e.g. mm) line 212 Add "the" before result and change "result" to "results." line 226 Change "event" to "events" line 278 Change "We" to lower case "we" line 319 Change" Researcher" to "Researchers" | We have revised this in the manuscript accordingly. |
| Besides areas for further research, the authors' conclusions should indicate implications for mitigating climate risks, such as avoiding the storage of water or ensuring that rooftop systems are airtight and household containers are covered to avoid breeding mosquitoes. | We have added the recommendation in manuscript line 290–293:  *“…For instance, practical strategies to address drought-related dengue risks include discouraging open water storage, sealing rooftop water systems, and covering household containers to minimize mosquito breeding and reduce the risk of dengue…”* |

Reviewer #6

| Reviewer’s comment | Author’s response |
| --- | --- |
| 1. Rationale for Selecting the Models: This study utilized functions to control environmental factors, considering various related factors such as seasonality, environmental influences, and random variables. However, the rationale for selecting this model raises the question of how confident we can be that the chosen model (GAMM) is the optimal one. To determine the optimal analytical methodology for examining the association between EWEs and dengue for this dataset, how can this confidence be justified? | Thank you very much for your reviews and comments. We have addressed your comments below:  We developed our model based on prior knowledge and exploratory analysis. Previous literature has demonstrated that climate factors exhibit non-linear effects on dengue transmission. GAMM were chosen because it is highly effective for capturing non-linear relationships due to the use of smooth function and provide flexibility for exploratory analysis. Also, dengue is strongly influenced by seasonality. To incorporate this, GAMM allows us to incorporate smooth terms to adjust for seasonal trends and other environmental factors. In addition, GAMM allows the inclusion of random effects to account for variability across locations, making them particularly suitable for our dataset. To ensure that the chosen model was the optimal choice for this dataset, we used generalised cross-validation to assess the predictive performance of the model, ensuring its robustness for the given dataset. However, while GAMM were determined to be the most appropriate choice for this study, we acknowledge that no single model is universally optimal. |
| 2. Smoothing Spline with 3 df: Your study employs a smoothing spline with 3 degrees of freedom based on a previous study conducted in Yogyakarta Province, Indonesia, which used Generalized Linear Regression Models (DOI:10.1371/journal.pone.0152688). Additionally, alternative methods, such as cross-basis functions, were also applied. How confident are you that these methods are the most appropriate for the dataset used in this study? | The selection of these methods was carefully considered based on prior literature, the mechanisme of climate effect on dengue as well as the characteristics of our dataset. The decision to use 3 degrees of freedom for the smoothing spline was based on evidence from previous research indicating that the relationship between temperature, precipitation, and dengue risk is non-linear, typically following an inverse U-shaped curve. This degree of freedom balances interpretability and model fit, ensuring that the model captures the complexity of the association without overfitting.  Additionally, cross-basis functions were employed to provide a more nuanced representation of both the immediate and lagged effects of climate variables on dengue transmission. This approach is essential, as the impact of climate on dengue dynamics often manifests after a delay due to its influence on the host, vector, and virus. Cross-basis functions effectively capture these delayed and non-linear effects while simultaneously accounting for interactions across different lags. To ensure the robustness of our findings, we conducted extensive sensitivity analyses. These included testing alternative specifications for the cross-basis functions. The results consistently supported the conclusions presented in the manuscript, demonstrating that our approach is both reliable and appropriate for examining the complex relationships between climate variables and dengue transmission. |
| 3. Definition of Heatwave: How confident can you be that the definition of heatwave used in this study aligns with the conditions in the areas studied and effectively represents its actual impact across all locations? | There is no universal definition for heatwave. Typically, it is often described as a period of consecutive days with temperatures surpassing a defined threshold. This threshold can be absolute (grounded in specific temperature values) or relative (based on the temperature within the community). In this research, heatwaves were defined using relative thresholds, which consider each location’s own long-term daily mean temperature. This method enables regional adaptation by comparing temperature to the norm for each specific location. In this study, a heatwave was characterized by two or more consecutive days with a daily mean temperature surpassing the 95th percentile of its distribution in each specific location. To ensure the robustness and transparency of our findings, we conducted multiple sensitivity analyses using alternative heatwave definitions, addressing the lack of a universally accepted definition. These analyses indicated that the findings were generally robust to changes in the heatwave definition. |
| 4. Managing Overfitting: How did you address the impact of overfitting when conducting multiple sensitivity analyses? | To manage overfitting, we developed our model based on a thorough review of the literature on the mechanisms through which climate impacts dengue, combined with extensive testing to ensure robustness through multiple sensitivity analyses. To achieve an optimal balance between model complexity and fit, we used the Generalized Cross-Validation (GCV) score to identify the minimal adequate fit for the model, avoiding overfitting while maintaining the ability to capture key relationships in the data. |
| 5. Statistical Reporting: The statistical reporting should include measures of size and/or distribution. For example, in lines 168-169, only the mean annual dengue case count is shown. These details should be more comprehensive. | We have revised the manuscript accordingly in line 186–188 and in Table 1.  *“…The Philippines reported the highest mean annual dengue cases [196,719 cases (SD=93,283)], followed by Vietnam [121,822 cases (SD=81748)] and Indonesia [112,156 cases (SD=42431)]….”* |
| 6. Assessment Results: The results from the assessment between the independent and dependent variables should comprehensively present the magnitude and distribution while clearly indicating statistical significance levels. | We have ensured that the results comprehensively present the magnitude and distribution of the relationships between the independent and dependent variables. We have also explicitly highlighted the levels of statistical significance to improve clarity and interpretability. |
| 7. Reporting Dengue Cases: Why was only RR used to report dengue case numbers? | Because the study use GAMM model with the quasi-Poisson distribution, which are commonly used for count data and are built with a log-link function. The output of this model is the Relative Risk (RR), derived from the exponentiation of the model coefficients. This measure expresses the change in dengue risk relative to a reference condition, making it both statistically appropriate and epidemiologically meaningful. |
| 8. Consistency of Terminology: Ensure consistent use of terms. For example, in line 184, the term "dengue relative" is used, in line 194, "only dengue," and in line 203, "dengue risk." Consistency in terminology would enhance clarity. | We have made changes in the manuscript accordingly. |
| 9. Abstract and Conclusions: The abstract and conclusions should be clearer and more robust. Recommendations for future research should build on this study, and practical implications for dengue prevention and control should be more explicitly addressed. | We have addressed your comment accordingly in manuscript line 54–57 and line 338–339:  line 54–57: *“…The findings highlight the need for public health interventions to mitigate the potential dengue risks posed by EWEs in the context of climate change in SEA. Future research should investigate the factors influencing variations in the EWE-dengue association across the region to support the development of tailored, location-specific mitigation and prevention strategies…”*  line 338–339: *“…The findings highlight the need for public health interventions to mitigate the potential dengue risks posed by EWEs in the context of climate change in SEA…”* |
| 10. Appendix Formatting: Table headers should be included on every page of the appendix. | We have added the table headers in appendix accordingly. |
| 11. I do not see any mention of the ethical considerations in the manuscript. I am not certain whether any identifiable personal data was collected (even if it was not utilized or reported in this study). Since this study involves data collected from multiple countries, if no identifiable personal data or other ethically sensitive information was collected, it should be explicitly stated in the manuscript. | We have added ethical consideration in the manuscript line 116–118  *“…The dengue dataset used in this study was fully anonymized prior to access and analysis. All identifying information was removed to ensure the privacy and confidentiality of individuals….”* |
